# Novel sunprotection interventions to prevent skin cancer: A randomized study targeting Danes going on vacation to destinations with high UV index

**DOI:** 10.1371/journal.pone.0244597

**Published:** 2020-12-31

**Authors:** Brian Køster, Mia N. Nielsen, Karina Kreipke Vester, Peter Dalum

**Affiliations:** 1 Department of Prevention and Information, Danish Cancer Society, Denmark; 2 Roskilde University Center, Roskilde, Denmark; Rutgers Cancer Institute of New Jersey, UNITED STATES

## Abstract

**Background:**

In Denmark, 16,500 cases of melanoma and keratinocyte cancers were registered in 2015, of which 90% could have been avoided by behavioral changes. We aimed to test novel interventions in a randomized design. The interventions targeted Danes going on vacation to high UVI destinations aiming to decrease sunburn by increasing use of sun protection to prevent skin cancer in the Danish population.

**Methods:**

We report a randomized behavioral intervention during May-Dec 2018 with 1548 Danish adults on vacation in 2018 for a period of 1–3 weeks. The study population was population-based and aged 18–65 years. We tested two protection routines against minimal intervention control group (2-by2-factorial design): 1) Avoidance of the sun during peak hours and shade, use of the UV-index and planning of indoor/outdoor activity respectively and, 2) Coverage by increasing use of the hat advice and increasing sunscreen amount by application routine. Outcome was use of protection and sunburn.

**Results:**

There were no differences in sunburn prevalence between intervention and control groups. Protection routine 1 and 2 both increased the overall protection score compared to non-users. Protection routine 1 increased the reported use of shade and decreased time exposed in the sun. Protection routine 2 increased the use of hat and sunscreen amount.

**Conclusion:**

Simple measures can help avoid the majority of one of the most widespread cancers worldwide. Vacations to high UVI destinations is a major influence on the annual Danish UV-exposure. We influenced travelers to protect themselves better and to increase sun protection behavior.

## Introduction

Incidence of melanoma and keratinocyte skin cancer have increased for decades especially in Caucasian populations **[1]**. Annually 16.500 Danes are diagnosed with a skin cancer and 300 die from skin cancer **[2]**. Exposure to ultraviolet radiation (UVR) from the sun and from artificial sources is the main risk factor **[3]**, however 90% of skin cancers could be avoided by behavioral changes **[4, 5]**. Vacations to destinations with a higher UV index (UVI) than in Denmark, constitutes a large proportion of Danes annual UVR exposure **[6**–**10]**.

The Danish Sun Safety Campaign communicates three sun protection messages to avoid skin cancer. They are all aiming to reduce exposure to UVR from the sun by applying: *Shade*, when the UVI reaches the daily maximum between 12 and 3 pm, *sunhat (and* clothes) and *sunscreen* for uncovered parts of the body. The sun protection messages are recommended in combination to be most effective. Avoidance of UVR by staying indoor obviously provides the best protection, especially in the middle of the day when UVR levels peaks. The level of sun protection can be described by the sun protection factor (SPF). Shade structures provide low protection against UVR (SPF = 2–5), due to reflection and diffuse UVR **[11]**. Textiles, like clothing and hats provides high protection of the covered areas(SPF > 50) **[12]**, while hats only provide low protection on shaded areas, like the face(SPF 2–4) **[13]**. Sunscreens are produced with a theoretical SPF up to 50 in Denmark (requires distribution of 2 mg/ml) which is categorized as high. Consumers apply approximately 0.5–1.0 mg/ml sunscreen, which is less than recommended. This results in the an effective protection of sunscreens labeled SPF 15 and SPF 30 of less than SPF 4 and SPF 5, respectively, which corresponds to low protection **[14]**. The Danish sun safety campaign previously achieved successful results including a decrease in the fraction of sunburn **[15]** and a decrease in use of sunbeds **[16]**; however, the fraction of sunburn in the population going on vacations to sunny destinations with high UVI was unchanged from 25% in 2008 to 27% in 2014 **[17]**.

Results from a large Norwegian study showed an OR of 1.7 for melanoma when comparing one or more annual vacations to high UVI destinations with less than one annual vacation **[18, 19]**. About half of the Danish population is going on a vacation to a sunny destination annually. Studies showed Danes going on vacation to destinations with high UVI received 43% of the annual UVR dose in just one week **[20, 21]** and found that all participants in the studies were sunburned determined by objective measurement **[22]**. When the UVI at a destination is increased compared to domestic levels, the radiation dose corresponding to the minimal erythemal dose of a person is reached in less time. Thus if sun protection or sun avoidance is not increased at destinations concordantly with increasing UVI the risk of sunburn will be higher at destinations with increasingly higher UVI as previously shown in a beachgoer study in Ohio **[23]**.

Danes travelling to destinations with higher UVI levels than Denmark are not having siestas like local populations do e.g. in Mediterranean countries. During 11am-3pm, 90% of Danes on vacation are outside more than an hour, and 41% are outside more than 3 hours **[24]**. The dose and intensity level of the radiation increases the risk of sunburns. Therefore prolonged times outdoors on vacations, especially to locations at lower latitudes increases the risk of sunburn. The sun protection message, hat, is the least used by the population as 80% of the Danes never wore a hat during their vacation and only 10% used it often or always **[24]**, which is unfortunate because 75% of non-melanoma skin cancers arise in the head, neck and scalp region **[25]**. Sunscreen is the sun protection message with the highest penetration in the population. In Australia, sunscreen was shown to reduce squamous cell skin cancer and melanoma under high UVR conditions **[26, 27]**; however the evidence for the protective effects of sunscreen against sunburn and melanoma is situation dependent **[28]**. Sunscreen with SPF 15 only lets 1/15 corresponding to ~7% of the erythemal UV radiation through; however, in real life there are many pitfalls when using sunscreen that can reduce its effectiveness, **[9, 14, 29, 30]**. The average sunscreen application of the Danes is less than 50% of the recommended amount **[14, 31]** and 50% of the Danes applying sunscreen used a sunscreen with insufficient sun protection factor (SPF<30) on their vacation and only 25% re-applied sunscreen sufficiently.

Our aim is to test novel interventions to decrease sunburn by increasing use of sun protection targeting Danes going on vacation to sunny destinations.

## Materials and methods

Approval by regional science ethical committees were waived by default according to Danish law as biological samples were not included in the study. The Danish data protection agency approved the access to participants in registries. Consent was obtained digital upon entry to the study. This trial was registered at clinicaltrials.gov with identifier: NCT03607578 **[32].** The authors confirm that all ongoing and related trials for this intervention are registered.

### Study design and participants

We conducted a randomized controlled trial with a 2-by-2 factorial design during May-Dec 2018 with 1548 Danish adults traveling on vacation in 2018 for a period of 1–3 weeks, recruited from the civil registration system. The study population was drawn in April to represent the Danish population aged 18–65 years. Participants were asked to sign up on a web page formula designed for the study. Concurrently they were asked about their contact details and holiday plans. Exclusion criteria for the study were incompatible cognitive skills to complete the intervention. Eligible for the study were persons living in Denmark going on vacation in May-December 2018 and having a smartphone. If participants informed they had plans to go on vacation but had not chosen time and destination yet, they were ad hoc contacted to retrieve updated vacation plans. Once a vacation plan was confirmed, their enrollment in the study was completed. Recruitment began in May and last vacation plan was scheduled in October. The first participant in the study went on vacation in the end of May and the last in the end of November. Participants were sent a link to a web questionnaire in the week they returned from vacation and if not completed a reminder was sent after 2 weeks. Data collection was completed in December. The study period was chosen as it includes all types of vacationers. The summer vacation constitutes the majority of the Danes annual vacation period. The majority of destinations visited by Danes in the summer has a higher ultraviolet radiation index compared to Denmark and the study period included most travel patterns, which change somewhat during seasons. In the summer holiday southern Europe is the main destination, but later in the year destinations closer to equator, e.g. Thailand increases. After enrollment, participants were randomly assigned to one of four experimental conditions. Three experimental groups received innovative low intensive intervention strategies for promoting sun protection practices during vacation–Protection Routine (1), Protection Routine (2) or both Protection Routines(1+2). The fourth experimental condition was a minimal treatment control group. All information material was sent by both email and physical letter. The primary outcomes of the trial was a reduction in frequency of sunburn by adherence to the current sun protection messages: use of shade, hats, protective clothing, and sunscreen as secondary outcomes. We hypothesized that the following two interventions would be able to reduce significantly the UVR exposure and risk of sunburns in Danes going on vacation to destinations with high UVI.

### Interventions

The Interventions builds on the Danish Sun Safety Campaigns theoretical framework, which is based on Theory of Planned Behavior, but also includes elements from Health Belief Model **[33]**. The interventions intended to provide knowledge, bring awareness and subsequently change attitudes, norms and behavior towards sun behavior.

Protection routine 1 focused on avoidance and included the rationale of the sun sun protection message, **[34]**, an activity planner, with suggestions to why a break from the sun is beneficial and how to plan outdoor high exposure activities before and after UVI maximum i.e. morning/early noon and afternoon/evening **[34]**, instructions to download of the UV-application ‘UVindeks’ for smartphones developed by the campaign and activating UV alert of geographic destination **[35]** and a skin type guide. The alert includes UV-information of the day and personal exposure duration information in response to skin type test. The app is available from GooglePlay and iOS store.

Protection routine 2 focused on coverage. The intervention included:

- A wide-brimmed hat to wear in the sun to test the use of hat when it is available **[36]**
- A newly developed instruction for correct application of sunscreen **[37]**

In Denmark people do not have a strong tradition of wearing hats and nor is there a large commercial availability of quality hats. By making hats available and using attractive quality, we aimed to increase this behavior. A study showed that a brief instruction to sunscreen application increased the provided protection **[37].** The sunscreen application instruction was based on a recently developed application instructions for use of sunscreen to counter the described problems and deficits of current sunscreen use. It describes every part of the body, the needed volume of sunscreen for that body part and application patterns. The package included a hat and the availability intended to increase the use. The hat was foldable for easy packing. The sunscreen flaws to avoid as well as the benefits from applying 2 layers of sunscreen was also included.

One group received both above mentioned interventions and will reveal potential dose response effects from level of protection. The control group will receive a minimal intervention with the current sun protection messages from the Danish Sun Safety Campaign. Intervention materials are available in supplementary materials.

All elements of the interventions were either previously available or tested for use in association to the campaign, used in other studies or pilot tested independently in small groups of 10–20 persons.

### Outcomes and variables

The primary success criteria of the interventions is a decrease in sunburn fraction in the intervention groups as compared to the control group in the post-intervention measurement. Sunburn was defined as any kind of erythema, discomfort, pain or blister with a duration of more than 12 hours after exposure to the sun. Secondary success criteria are increased awareness on the risk of skin cancer, increased use of protection (shade/clothes/hat/sunscreen) and decreased outdoor exposure when the UVI is highest (12-3pm.) between interventions and control group. Additionally, decrease in body-site specific sunburn by prevention method (e.g. hat use and sunburn in head region), increase in knowledge and perceived importance of sun protection. Participants were asked about country and location of their destination. The vacation destination was then assigned a UVI value according to time of year and UVI level retrieved from the Danish Meteorological Institute.

Sun protective behavior, exposure to UVR and sunburn was evaluated by use of a questionnaire validated by personal electronic UV-measurements **[6, 38, 39]**. This developed survey is published, publicly available and this tool has made it possible in this project to evaluate the skin cancer interventions in relation to exposure **[40]**, without tracking of development of skin cancer, which can take several years to develop after excessive UVR exposure. Additional information on scores and scales applied was previously described **[41]**. The short time period from intervention to evaluation minimizes recall bias.

### Sample size and randomization

**Fig 1:**
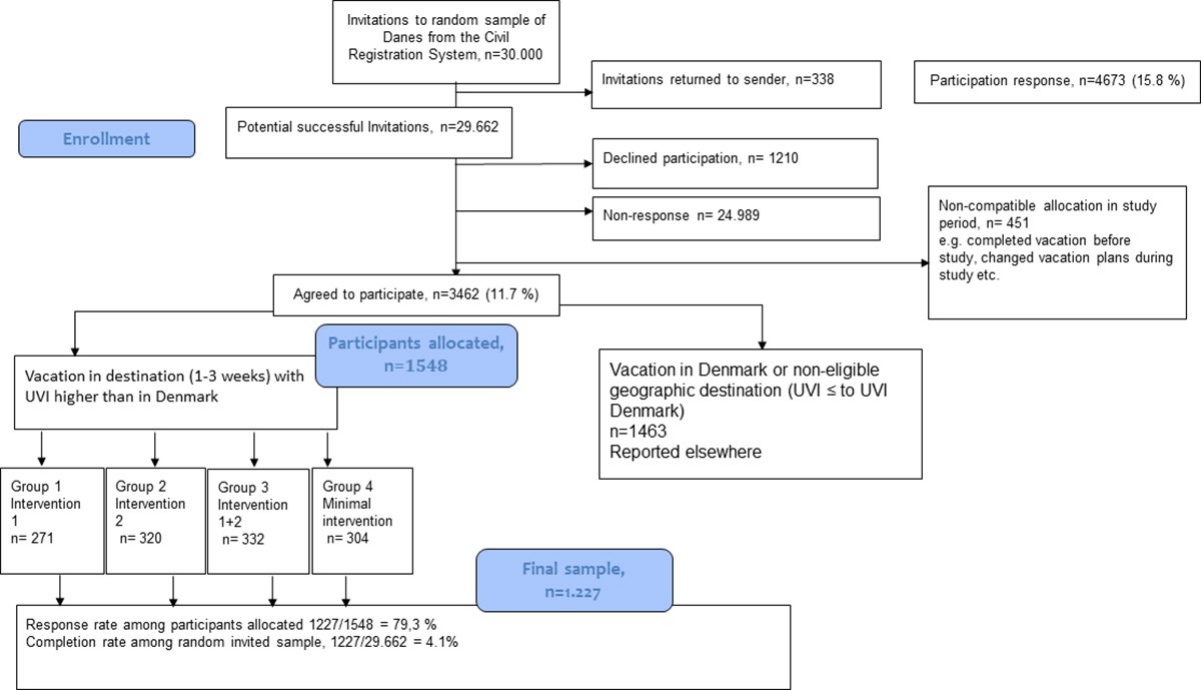
Flow diagram.

The final sample size and the recruitment process is described in Fig 1. The participants were randomized to one of the groups. The randomization procedure was an ad hoc procedure, because it was dependent on participants registering for the project and their vacation plans, which could e.g. change or be a last-minute deal. When signing up using the web formula described above participants were automatically randomized for an intervention group in the survey system, surveyXact. The randomization consisted of a question functioning as a lottery (numbers 1–40) where participants had to pick a random number, which was coded for an intervention group (1–4). The random sequence was numeric from 1 to 40 and intervention groups was assigned 10 numbers each and designed by BK. The intervention allocation was concealed from the participants. KLT enrolled and assigned participants to interventions based on the randomization. The participants were informed that they were to test the given sun protection messages of the campaign. Possible confounders includes age, gender, education, skin type, skin cancer history, moles, sun-seeking behavior and weather of destination **[28]**.

### Statistical methods

The number of participants to recruit was based on a power calculation, a pilot study and aimed for 1,980 participants **[40, 42].** The sample size yields 95% probability to detect a 10-percentage point drop from 50% to 40% in sunburn between an intervention group and the control group (α = 0.05, two-tailed). The number of invited participants to reach a final sample of ~2,000 are included in the flow diagram in the protocol. To obtain the final study sample size, we assumed 25% of Danes invited to participate will volunteer for the project **[40]**, >80% will use interventions and >90% will complete questionnaire **[40, 42]**.

Multiple logistic regression analysis was applied for analysis of differences in sunburn fraction. Analysis of variance (one-way ANOVA) was applied to analyze differences in sun protection score and UVR exposure score. Alpha-criterion: p = 0.05 (two-tailed). Intention-to-treat analysis was applied. To examine if low penetration of the interventions influenced the results we analysed users of the interventions vs non-users of the interventions i.e. including only participants who used the assigned intervention (per protocol), defined as minimal use of all the included elements. All parts of intervention use were analysed on a 5-point Likert-scale. Users of the interventions were defined as those answering any use (2–5 points) for all parts on intervention 1 (app, avoidance folder, activity guide, skin type guide) and intervention 2 (hat, coverage folder, sunscreen guide) respectively. The statistical software package SAS version 9.4 (SAS Institute, NC, USA) was used for the analyses.

## Results

Thirty thousand persons were invited of which 3,462 (11.7%) agreed to participate and 1,548 were eligible and had planned to go on a vacation to a destination with a higher UV-index in the study period than the average UV-index in Denmark (Fig 1). Those eligible were sent the intervention material. The final sample constituted of 1,227 persons who completed the questionnaire corresponding to a response rate among those allocated of 79.3%. Most participants from intervention group 3 completed and least from intervention group 1. Table 1 shows the distribution of demographic characteristics in the intervention groups. There were no differences between the groups on gender, age, skin type, region or education, though region was borderline significant. Table 2 shows the usage of interventions distributed on intervention groups and demographics. A high fraction of the intervention materials reached the participants (97%); including sun hats (95%). Usage of the intervention parts ranged between 65% for using the uv-index app and 88% for using information pamphlet about sunscreen and sunhat. More females used the uv-index app and the skin type guide. There were only minor regional variation, while persons with primary school education in general used interventions the least.

**Table 1:** Distribution of demographic characteristics in four armed randomized sample of 1227 Danes.

Characteristic (%) Total (n = 1227)	Total n	%	Intervention 1 (Group 1) n (%)	Intervention 2 (Group 2) n (%)	Intervention 1 + 2 (Group 3) n (%)	Minimal intervention /Control (Group 4) n (%)
Total	1227	100	271 (22.1)	320 (26.1)	332 (27.1)	304 (24.8)
Gender						
Male	388	31.6	74 (27.3)	106 (33.1)	110 (33.1)	98 (32.2)
Female	839	68.4	197 (72.7)	214 (66.9)	222 (66.9)	206 (67.8)
Age group						
18–24	210	17.1	47 (17.3)	59 (18.4)	56 (16.9)	48 (15.8)
25–34	233	19.0	49 (18.1)	58 (18.1)	64 (19.3)	62 (20.4)
35–44	278	22.7	64 (23.6)	72 (22.5)	72 (21.7)	70 (23.0)
45–54	320	26.1	71 (26.2)	88 (27.5)	92 (27.7)	69 (22.7)
55–65	186	15.2	40 (14.8)	43 (13.4)	48 (14.5)	55 (18.1)
Skin type						
I	117	9.5	31 (11.4)	28 (8.8)	33 (9.9)	25 (8.2)
II	755	61.5	152 (56.1)	203 (63.4)	204 (61.4)	196 (64.5)
III/ IV	355	28.9	88 (32.5)	89 (27.8)	95 (28.6)	83 (27.3)
Region						
Capital	473	38.5	105 (38.8)	136 (42.5)	140 (42.2)	92 (30.3)
Zealand	125	10.2	22 (8.1)	31 (9.7)	35 (10.5)	37 (12.2)
Northern Jutland	105	8.6	25 (9.2)	19 (5.9)	28 (8.4)	33 (10.9)
Central Jutland	276	22.5	56 (20.7)	68 (21.3)	70 (21.1)	82 (27.0)
Southern Denmark	248	20.2	63 (23.2)	66 (20.6)	59 (17.8)	60 (19.7)
Education						
Primary school	39	3.2	5 (1.8)	8 (2.5)	7 (2.1)	19 (6.3)
Secondary school	94	7.7	15 (5.5)	23 (7.2)	30 (9.0)	26 (8.6)
Vocational	207	16.9	50 (18.5)	54 (16.9)	63 (19.0)	40 (13.2)
Higher education (<2y)	136	11.1	32 (11.8)	36 (11.3)	36 (10.8)	32 (10.5)
Higher education (2–4½y)	439	35.8	100 (36.9)	119 (37.2)	108 (32.5)	112 (36.8)
Higher education (>4½y) Other	308 4	25.1 0.3	68 (25.1) 1 (0.4)	78 (24.4) 2 (0.6)	88 (26.5) 0 (0)	74 (24.3) 1 (0.3)
UVI at destination (mean;95% CI) Range (5.5–12.3)			7.99 (7.81–8.18)	8.27 (8.09–8.44)	8.01 (7.85–8.17)	8.11 (7.92–8.30)

P-values are for χ ^2^-test between factor levels and intervention groups.

**Table 2:** Distribution of usage of interventions by intervention group and demographic characteristics in a randomized sample of 1227 Danes.

	Receipt			Usage					
	Received material	App downloaded	Received hat	App	Shade pamphlet	Sunscreen / hat pamphlet	Sunscreen step-by-step guide	Activity planner	Skin type guide
Total	1227 (97)	1227 (30)	626 (95)	544 (65)	1227 (42)	1227 (47)	1227 (44)	1227 (33)	1227 (36)
Group									
1	264 (97.4)	166 (61.3)	N.A.	169 (70.7)	227 (83.8)	N.A.	N.A.	185 (68.3)	202 (74.5)
2	311 (97.2)	18 (5.6)	289 (94.4)	N.A.	N.A.	279 (87.2)	266 (83.1)	N.A.	N.A.
3	327 (98.5)	162 (48.8)	305 (95.3)	184 (60.5)	289 (87.05)	295 (88.9)	274 (82.5)	225 (67.8)	239 (72)
4	284 (93.4)	19 6.3)	N.A.	N.A.	N.A.	N.A.	N.A.	N.A.	N.A.
Gender	*p = 0*.*085*	***p<0*.*001***	*p = 0*.*607*	***p<0*.*001***	*p = 0*.*206*	*p = 0*.*855*	*p = 0*.*733*	*p = 0*.*1000*	*p = 0*.*064*
Male	370 (95.4)	**89 (22.9)**	193 (95.5)	**87 (53.4)**	153 (39.4)	183 (47.2)	168 (43.3)	193 (95.5)	125 (32.2)
Female	816 (97.3)	**276 (32.9)**	401 (94.6)	**266 (69.8)**	363 (43.3)	391 (46.6)	372 (44.3)	401 (94.6)	316 (37.7)
Age group	*p = 0*.*980*	*p = 0*.*941*	*p = 0*.*387*	*p = 0*.*392*	*p = 0*.*865*	*p = 0*.*494*	*p = 0*.*548*	*p = 0*.*377*	*p = 0*.*702*
15–24	203 (96.7)	61 (29.1)	106 (94.6)	55 (58.5)	87 (41.4)	98 (46.7)	91 (43.3)	71 (33.8)	84 (40)
25–34	225 (96.6)	72 (30.9)	112 (94.9)	63 (62.4)	91 (39.1)	104 (44.6)	98 (42.1)	65 (27.9)	80 (34.3)
35–44	269 (96.8)	85 (30.6)	135 (97.8)	85 (70.8)	119 (42.8)	130 (46.8)	128 (46)	99 (35.6)	95 (34.2)
45–54	308 (96.3)	96 (30)	160 (94.1)	98 (66.7)	138 (43.1)	162 (50.6)	149 (46.6)	109 (34.1)	114 (35.6)
55–65	181 (97.3)	51 (27.4)	81 (92.1)	52 (63.4)	81 (43.6)	80 (43)	74 (39.8)	66 (35.5)	68 (36.6)
Skin type	*p = 0*.*805*	*p = 0*.*543*	*p = 0*.*792*	*p = 0*.*923*	*p = 0*.*280*	*p = 0*.*726*	*p = 0*.*780*	*p = 0*.*640*	*p = 0*.*950*
I	113 (96.6)	40 (34.2)	57 (96.6)	35 (67.3)	55 (47)	53 (45.3)	51 (43.6)	43 (36.3)	42 (35.9)
II	728 (96.4)	221 (29.3)	370 (94.9)	210 (64.8)	305 (40.4)	360 (47.7)	338 (44.8)	246 (32.6)	269 (35.6)
III/ IV	345 (97.2)	104 (29.3)	167 (94.4)	108 (64.3)	156 (43.9)	161 (45.4)	151 (42.5)	121 (34.1)	130 (36.6)
Region	*p = 0*.*132*	*p = 0*.*863*	*p = 0*.*646*	*p = 0*.*853*	*p = 0*.*886*	*p = 0*.*252*	*p = 0*.*234*	*p = 0*.*383*	*p = 0*.*502*
Capital	458 (96.83)	145 (30.7)	255 (95.9)	144 (63.7)	198 (41.9)	234 (49.5)	223 (47.2)	147 (31.1)	179 (37.8)
Zealand	125 (100)	32 (25.6)	58 (92.1)	29 (61.7)	51 (40.8)	62 (49.6)	56 (44.8)	44 (35.2)	39 (31.2)
Northern Jutland	99 (94.3)	32 (30.5)	45 (95.7)	33 (64.7)	47 (44.8)	40 (38.1)	37 (35.2)	35 (33.3)	42 (40)
Central Jutland	264 (95.7)	81 (29.4)	125 (93.3)	72 (654.3)	111 (40.2)	126 (45.7)	117 (42.4)	89 (32.3)	93 (33.7)
Southern Denmark	240 (96.8)	75 (30.2)	111 (95.7)	75 (69.4)	109 (44)	112 (45.2)	107 (43.2)	95 (38.3)	88 (35.5)
Education	***p = 0*.*078***	***p<0*.*001***	*p = 0*.*264*	*p = 0*.*072*	*p = 0*.*143*	*p = 0*.*534*	*p = 0*.*301*	*p = 0*.*293*	***p = 0*.*037***
Primary school	**36 (92.3)**	**3 (7.7)**	13 (86.7)	4 (44.4)	11 (28.2)	13 (33.3)	10 (25.6)	9 (23.1)	**9 (23.1)**
Secondary school	**201 (97.1)**	**66 (31.9)**	112 (98.3)	63 (60)	98 (47.3)	101 (48.8)	97 (46.9)	76 (36.7)	**91 (44)**
Vocational	**89 (94.7)**	**23 (24.5)**	44 (89.8)	20 (51.3)	37 (39.4)	40 (42.6)	40 (42.6)	29 (30.9)	**27 (28.7)**
Higher education (<2y)	**127 (93.4)**	**43 (31.6)**	66 (95.7)	41 (66.1)	58 (42.7)	63 (46.3)	61 (44.9)	43 (31.6)	**51 (37.5)**
Higher education (2–4½y)	**431 (98.2)**	**115 (26.2)**	208 (95)	126 (65.6)	177 (40.3)	206 (46.9)	190 (43.3)	140 (31.9)	**152 (34.6)**
Higher education (>4½y)	**298 (96.8)**	**115 (37.3)**	149 (94.3)	99 (72.8)	135 (43.8)	150 (48.7)	141 (45.8)	113 (36.7)	**111 (36)**
Other	**4 (100)**	**0 (0)**	2 (100)	0 (0)	0 (0)	1 (25)	1 (25)	0 (0)	**0 (0)**

Values are n (%). P-values are for χ^2^-test between factor levels and intervention. N.A (Not applied)

Table 3 shows distribution of sunburn total and sunburn specific sites (dichotomized). There were no differences in total sunburn between the 4 intervention groups, neither among those who received the interventions, however more users of intervention 2 were sunburned compared to non-users. For site-specific sunburn, again, there were no differences between the 4 intervention groups or receivers/non-receivers of the interventions. Fewer users of intervention 1 were sunburned on the arms and more users of intervention 2 were sunburned on the shoulders. In an adjusted logistic regression analysis, there were no significant differences between the 4 intervention groups in total sunburn. Receivers of intervention 1 and 2 experienced no significant differences in sunburn compared to non-receivers. In general, a larger fraction of males, young people and pale skin types were sunburned. There was an increased risk of sunburn among persons with secondary school and vocational education compared to higher education of more than 4½ years of duration. Additionally, the risk of sunburn increased with 1.22 (1.11–1.34) for each increasing level of UVI.

**Table 3:** Logistic regression analysis of dichotomous sunburn by intervention/Intervention usage (left). Distribution of total sunburn and sunburn specific site on demographic variables and intervention groups (right).

Characteristic (%) Total (n = 1227)	n (%)	OR (95% CI)	Sunburn total	Sunburn Head / Neck	Sunburn Hands /Feet	Sunburn Shoulder	Sunburn Legs	Sunburn Arms	Sunburn Back torso	Sunburn Front torso
Total	1227 (100)		681 (56)	376 (31)	113 (9.2)	401 (32.7)	192 (15.7)	238 (19.4)	290 (24)	224 (18)
Intervention Group		*p* = 0.872	*p = 0*.*56*	*p = 0*.*527*	*p = 0*.*724*	*p = 0*.*439*	*p = 0*.*209*	*p = 0*.*438*	*p = 0*.*409*	*p = 0*.*719*
1	271 (22.1)	1.06 (0.70–1.60)	146 (53.9)	76 (28.0)	23 (8.5)	80 (29.5)	32 (11.8)	45 (16.6)	55 (20.3)	44 (16.2)
2	320 (26.1)	1.01 (0.68–1.49)	182 (56.9)	95 (29.7)	26 (8.1)	112 (35)	54 (16.9)	59 (18.4)	84 (26.3)	63 (19.7)
3	332 (27.1)	1.15 (0.78–1.70)	192 (57.8)	111 (33.4)	32 (9.6)	114 (34.3)	59 (17.8)	69 (20.8)	79 (23.8)	63 (19.0)
4	304 (24.8)	1.00 (ref)	161 (53.0)	94 (30.9)	32 (10.5)	95 (31.3)	47 (15.5)	65 (21.3)	72 (23.7)	54 (17.8)
Intervention 1		*p = 0*.*476*	*p = 0*.*70*	*p = 0*.*78*	*p = 0*.*92*	*p = 0*.*71*	*p = 0*.*60*	*p = 0*.*67*	*p = 0*.*25*	*p = 0*.*65*
Received	603 (49)	1.10 (0.84–1.45)	338 (56.1)	187 (31.0)	55 (9.1)	194 (32.2)	91 (15.0)	114 (18.9)	134 (22.2)	107 (17.7)
Not received	624 (51)	1.00 (ref)	343 (55.0)	189 (30.3)	58 (9.3)	207 (33.2)	101 (16.2)	124 (19.9)	156 (25.0)	117 (18.8)
Intervention 2		*p = 0*.*731*	*p = 0*.*16*	*p = 0*.*44*	*p = 0*.*69*	*p = 0*.*12*	*p = 0*.*08*	*p = 0*.*82*	*p = 0*.*23*	*p = 0*.*30*
Received	652 (53)	1.05 (0.80–1.38)	374 (57.4)	206 (31.6)	58 (8.9)	226 (34.7)	113 (17.3)	128 (19.6)	163 (25.0)	126 (19.3)
Not received	575 (47)	1.00 (ref)	307 (53.4)	170 (29.6)	55 (9.6)	175 (30.4)	79 (13.7)	110 (19.1)	127 (22.1)	98 (17.0)
Intervention 1		*p = 0*.*128*	*p = 0*.*95*	*p = 0*.*32*	*p = 0*.*93*	*p = 0*.*15*	*p = 0*.*62*	***p = 0*.*01***	*p = 0*.*76*	*p = 0*.*99*
Used	246 (20)	1.30 (0.93–1.83)	137 (55.7)	206 (28.1)	48 (9.8)	71 (28.9)	36 (14.6)	**34 (13.8)**	60 (24.4)	45 (18.3)
Not used	981 (80)	1.00 (ref)	544 (55.5)	170 (31.3)	65 (8.8)	330 (33.6)	156 (15.9)	**204 (20.8)**	230 (23.5)	179 (18.3)
Intervention 2		*p = 0*.*081*	***p = 0*.*03***	*p = 0*.*46*	*p = 0*.*56*	***p = 0*.*01***	*p = 0*.*10*	*p = 0*.*66*	*p = 0*.*21*	*p = 0*.*20*
Used	490 (40)	1.28 (0.97–1.68)	**291 (59.4)**	156 (31.8)	55 (9.1)	**180 (36.7)**	87 (17.8)	98 (20)	125 (25.5)	98 (20.0)
Not used	737 (60)	1.00 (ref)	**390 (52.9)**	220 (29.9)	32 (10.5)	**221 (30.0)**	105 (14.3)	140 (19)	165 (22.4)	126 (17.1)
Gender		*p<*0.001	*p = 0*.*002*	*p<0*.*0001*	*p = 0*.*005*	*p = 0*.*676*	*p = 0*.*057*	*p = 0*.*373*	*p = 0*.*696*	*p = 0*.*002*
Male	388 (32)	1.00 (ref)	241 (62.1)	159 (41.0)	49 (12.6)	130 (33.5)	72 (18.6)	81 (20.9)	89 (23.0)	90 (23.2)
Female	839 (68)	0.48 (0.36–0.65)	440 (52.4)	217 (25.9)	64 (7.6)	271 (32.3)	120 (14.3)	157 (18.7)	201 (24.0)	134 (16.0)
Agegroup		*p<0*.*001*	*p<0*.*001*	*p<0*.*0001*	*p = 0*.*160*	*p<0*.*0001*	*p<0*.*0001*	*p<0*.*0001*	*p<0*.*0001*	*p<0*.*0001*
18–24	210 (17)	3.68 (2.08–6.50)	150 (71.4)	81 (38.6)	18 (8.6)	99 (47.1)	47 (22.4)	51 (24.3)	66 (31.4)	54 (25.7)
25–34	233 (19)	5.59 (3.39–9.20)	167 (71.7)	100 (42.9)	30 (12.9)	106 (45.5)	56 (24.0)	76 (32.6)	73 (31.3)	48 (20.6)
35–44	278 (23)	2.72 (1.74–4.27)	155 (55.8)	80 (28.8)	27 (9.7)	86 (30.9)	43 (15.5)	47 (16.9)	68 (24.5)	55 (19.8)
45–54	320 (26)	1.99 (1.28–3.10)	152 (47.5)	84 (26.3)	27 (8.4)	83 (25.9)	39 (12.2)	46 (14.4)	69 (21.6)	54 (16.9)
55–65	186 (15)	1.00 (ref)	57 (30.7)	31 (16.7)	11 (9.7)	27 (14.5)	7 (3.8)	18 (9.7)	14 (7.5)	13 (7.0)
Skintype		*p<0*.*001*	*p = 0*.*001*	*p<0*.*0001*	*p<0*.*001*	*p<0*.*0001*	*p<0*.*0001*	*p<0*.*0001*	*p<0*.*0001*	*p<0*.*001*
I	117 (10)	6.70 (3.78–11.87)	92 (78.6)	57 (48.7)	15 (12.8)	61 (52.1)	30 (25.6)	45 (38.5)	37 (31.6)	20 (17.1)
II	755 (62)	2.72 (1.74–4.27)	455 (60.3)	265 (35.1)	84 (11.1)	274 (36.3)	137 (18.2)	160 (21.2)	198 (26.2)	163 (21.6)
III/ IV	355 (29)	1.00 (ref)	134 (37.8)	54 (15.2)	14 (3.9)	66 (18.6)	25 (7.0)	33 (9.3)	55 (15.5)	41 (11.6)
Region		*p = 0*.*452*	*p = 0*.*20*	*p = 0*.*105*	*p = 0*.*850*	*p = 0*.*837*	*p = 0*.*491*	*p = 0*.*045*	*p = 0*.*197*	*p = 0*.*948*
Capital	473 (39)	0.80 (0.55–1.17)	268 (56.7)	139 (29.4)	44 (9.3)	152 (32.1)	83 (17.6)	103 (21.8)	112 (23.7)	83 (17.6)
Zealand	125 (10)	0.62 (0.37–1.04)	57 (45.6)	28 (22.4)	10 (8.0)	36 (28.8)	14 (11.2)	14 (11.2)	21 (16.8)	21 (16.8)
Northern Jutland	105 (9)	0.64 (0.37–1.12)	58 (55.2)	30 (28.6)	7 (6.7)	35 (33.3)	15 (14.3)	21 (20.0)	32 (30.5)	21 (20.0)
Central Jutland	276 (22)	0.86 (0.57–1.30)	160 (58.0)	93 (33.7)	27 (9.8)	92 (33.3)	43 (15.6)	60 (21.7)	67 (24.3)	53 (19.2)
Southern Denmark	248 (20)	1.00 (ref)	138 (55.7)	86 (34.7)	25 (10.1)	86 (34.7)	37 (14.9)	40 (16.1)	58 (23.4)	46 (18.6)
Education		*p = 0*.*017*	*p = 0*.*001*	*p = 0*.*006*	*p = 0*.*567*	*p<0*.*0001*	*p<0*.*001*	*p = 0*.*001*	*p<0*.*0001*	*p<0*.*0001*
Primary school	39 (3)	0.80 (0.36–1.78)	19 (48.7)	9 (23.1)	6 (15.4)	11 (28.2)	10 (25.6)	6 (15.4)	10 (25.6)	8 (20.5)
Secondary school	207 (8)	1.74 (0.99–3.04)	151 (73.0)	79 (38.2)	23 (11.1)	101 (48.8)	49 (23.7)	19 (20.2)	78 (37.7)	61 (29.5)
Vocational	94 (17)	1.33 (0.73–2.40)	57 (60.6)	34 (36.2)	11 (11.7)	34 (36.2)	20 (21.3)	60 (29.0)	28 (29.8)	22 (23.4)
Higher education (<2y)	136 (11)	0.80 (0.36–1.78)	73 (53.7)	43 (31.6)	12 (8.8)	36 (26.5)	18 (13.2)	19 (14.0)	38 (27.9)	30 (22.1)
Higher education (2–4½y)	439 (36)	0.76 (0.50–1.16)	200 (45.6)	106 (24.2)	35 (8)	111 (25.3)	46 (10.5)	66 (15.0)	72 (16.4)	60 (13.7)
Higher education (>4½y)	308 (25)	1.00 (ref)	179 (58.1)	104 (33.8	26 (8.4)	106 (34.4)	48 (15.6)	67 (21.8)	62 (20.1)	42 (13.6)
UV-Index (pr. Level increase)	1227 (100)	1.22 (1.11–1.34)								
Exposure scale (pr. Level increase)	1227 (100)	1.06 (1.02–1.11))								
Self-reported clear sky weather (pr. Level decrease)	1227 (100)	0.80 (0.66–97)								

Multiple Logistic Regression Model included gender, age group, skintype, region, education, self-reported weather, UV-Index at location and exposure scale. p-values are for tests for variation for differences between factor levels.

In Table 4 is shown the adjusted sun behavior related mean (CI 95%) scores distributed on intervention groups. There were no significant differences on the sunburn scale (sum of sunburned areas and severity) between the 4 intervention groups. Use of intervention 1 (vs no use) showed a higher score on the overall protection scale and similarly use of intervention 2 (vs no use) showed a higher score too. There were no differences between the groups regarding UV exposure. The importance of sun protection when participants are on vacation to destination with higher UVI compares to Denmark was regarded higher among users of both intervention 1 and 2 (vs no use). Sunscreen ability scale (amount, reapplication, time of application, factor) was borderline significant in users of intervention 2. There were no difference between groups in knowledge of UVR and Melanoma. Group 2 showed a higher use of sunscreen on body parts and similarly for users of intervention 2. Receivers and users of intervention 1 scored lower on outdoor exposure time between 11 and 15 compared to nonusers. Users of intervention 2 scored higher on time-fraction wearing sunscreen (minimum SPF 15) compared to nonusers. There were no difference between groups for using long sleeved clothing or long pants. There was no differences between groups in cap use. Group 2 and 3 showed significantly higher use of wide brimmed hat, which was part of these interventions. Group1, but not group 3 showed higher use of shade, which was part of these interventions. Combined both receivers and users of intervention 1 used shade a larger fraction of the time. Staying inside scored higher in both group 1 and 3, for both receivers and users of intervention 1.

**Table 4:** Sun behavior related adjusted mean (95% CI) scores distributed on intervention group in a random sample.

	Outdoor11_15	SPF15	Long sleeves	Long pants	Cap	Wide-brimmed hat	Shade	Stay Inside
Group	*p =* 0.461	*p =* 0.199	*p =* 0.767	*p =* 0.198	*p =* 0.301	*p<*0.001	*p =* 0.009	*p =* 0.032
1: I1	2.51 (2.30–2.72)	2.31 (2.12–2.50)	0.79 (0.57–1.01)	0.49 (0.32–0.67)	0.99 (0.75–1.23)	**0.35 (0.11–0.59)**	**2.02 (1.83–2.22)**	**0.98 (0.79–1.17)**
2: I2	2.57 (2.37–2.78)	2.36 (2.18–2.54)	0.92 (0.49–0.92)	0.38 (0.21–0.56)	0.83 (0.59–1.07)	**0.92 (0.68–1.15)**	**1.77 (1.58–1.96)**	**0.79 (0.60–0.98)**
3: I1+I2	2.47 (2.26–2.68)	2.35 (2.17–2.53)	0.75 (0.53–0.96)	0.49 (0.32–0.66)	0.83 (0.59–1.07)	**0.93 (0.70–1.17)**	**1.86 (1.67–2.05)**	**0.97 (0.78–1.16)**
4: Control	2.58 (2.38–2.79)	2.21 (2.03–2.40)	0.76 (0.55–0.98)	0.52 (0.35–0.69)	0.90 (0.67–1.14)	**0.51 (0.27–0.74)**	**1.83 (1.64–2.02)**	**0.89 (0.70–1.08)**
Intervention 1	***p =* 0.125**	*p =* 0.460	*p =* 0.542	*p =* 0.334	*p =* 0.566	*p =* 0.386	***p =* 0.012**	***p =* 0.009**
Received	**2.49 (2.30–2.68)**	2.32 (2.15–2.49)	0.77 (0.57–0.97)	0.50 (0.34–0.66)	0.91 (0.69–1.13)	0.64 (0.41–0.86)	**1.94 (1.76–2.12)**	**0.98 (0.81–1.16)**
Not received	**2.58 (2.39–2.77)**	2.28 (2.12–2.45)	0.73 (0.54–0.93)	0.45 (0.29–0.61)	0.87 (0.65–1.09)	0.70 (0.47–0.92)	**1.80 (1.63–1.98)**	**0.84 (0.67–1.01)**
Intervention 2	*p =* 0.678	*p =* 0.079	*p =* 0.397	*p =* 0.162	*p =* 0.087	***p<*0.001**	***p =* 0.041**	*p =* 0.317
Received	2.52 (2.33–2.52)	2.35 (2.18–2.52)	0.72 (0.53–0.92)	0.44 (0.28–0.60)	0.83 (0.61–1.05)	**0.93 (0.71–1.15)**	**1.81 (1.63–1.98)**	0.88 (0.70–1.05)
Not received	2.55 (2.36–2.74)	2.26 (2.09–2.43)	0.78 (0.58–0.97)	0.51 (0.35–0.67)	0.94 (0.73–1.16)	**0.43 (0.22–0.65**	**1.92 (1.74–2.09)**	0.93 (0.76–1.11)
Intervention 1	***p =* 0.004**	*p =* 0.714	*p =* 0.881	*p =* 0.516	*p =* 0.143	*p =* 0.541	***p<*0.001**	*p =* 0.066
Used	**2.36 (2.14–2.58)**	2.32 (2.13–2.52)	0.76 (0.53–0.99)	0.51 (0.39–0.69)	1.00 (0.74–1.25)	0.71 (0.45–0.97)	**2.07 (1.87–2.27)**	1.01 (0.81–1.22)
Not used	**2.57 (2.38–2.75)**	2.30 (2.14–2.46)	0.75 (0.56–0.94)	0.47 (0.32–0.62)	0.87 (0.66–1.08)	0.66 (0.45–0.87)	**1.83 (1.66–2.00)**	0.89 (0.72–1.06)
Intervention 2	*p =* 0.220	***p<*0.001**	*p =* 0.560	*p =* 0.557	*p =* 0.863	***p<*0.001**	*p =* 0.373	*p =* 0.503
Used	2.49 (2.30–2.69)	**2.46 (2.29–2.63)**	0.77 (0.57–0.98)	0.46 (0.29–0.62)	0.88 (0.66–1.11)	**1.11 (0.89–1.32)**	1.84 (1.65–2.02)	0.88 (0.70–1.06)
Not used	2.56 (2.38–2.75)	**2.21 (2.05–2.37)**	0.74 (0.55–0.93)	0.48 (0.33–0.64)	0.89 (0.68–1.11)	**0.41 (0.20–0.62)**	1.88 (1.71–2.06)	0.92 (0.79–1.09)
**Sun behavior related adjusted mean (OR (95% CI)) scores distributed on intervention group in a random sample.**								
	Burn Scale	Protection scale	Exposure scale	Important to protect abroad scale	Sunscreen ability scale		Knowledge scale	Sunscreen body part scale
Group	*p* = 0.286	*p* = 0.210	*p* = 0.653	*p* = 0.399	*p* = 0.091		*p* = 0.913	*p* = 0.350
1: I1	1.95 (1.44–2.48)	7.94 (7.22–8.66)	12.86 (12.14–13.57)	8.51 (8.03–8.98)	5.61 (5.33–5.89)		13.09 (12.28–13.90)	14.22 (13.38–15.05)
2: I2	2.24 (1.73–2.75)	7.75 (7.04–8.46)	13.09 (12.39–13.79)	8.41 (7.94–8.87)	5.73 (5.45–6.01)		13.24 (12.45–13.04)	14.61 (13.80–15.43)
3: I1+I2	2.34 (1.83–2.85)	8.18 (7.48–8.89)	12.77 (12.07–13.47)	8.61 (8.14–9.07)	5.50 (5.23–5.78)		13.23 (12.43–13.02)	14.11 (13.30–14.92)
4: Control	2.27 (1.76–2.78)	7.63 (6.92–8.33)	12.85 (12.15–13.55)	8.30 (7.84–8.77)	5.47 (5.19–5.75)		13.05 (12.25–12.85)	14.10 (13.28–14.93)
Intervention 1	*p* = 0.580	*p* = 0.057	*p* = 0.365	*p* = 0.123	*p* = 0.426		*p* = 0.962	*p* = 0.326
Received	2.16 (1.69–2.64)	8.06 (7.41–8.72)	12.80 (12.15–13.44)	8.56 (8.12–8.99)	5.54 (5.28–5.80)		13.15 (12.41–13.89)	14.13 (13.38–14.89)
Not received	2.24 (1.77–2.71)	7.68 (7.03–8.33)	12.98 (12.34–13.62)	8.35 (7.92–8.78)	5.60 (5.35–5.85)		13.14 (12.41–13.87)	14.37 (13.62–15.11)
Intervention 2	*p =* 0.226	*p =* 0.359	*p =* 0.715	*p =* 0.443	*p =* 0.368		p = 0.475	*p =* 0.407
Received	2.30 (1.83–2.77)	7.96 (7.30–8.61)	12.93 (12.28–13.58)	8.50 (8.07–8.93)	5.61 (5.35–5.87)		13.23 (12.49–13.97)	14.36 (13.60–15.11)
Not received	2.12 (1.65–2.59)	7.77 (8.12–8.42)	12.86 (12.22–13.50)	8.40 (7.97–8.82)	5.54 (5.28–5.79)		13.07 (12.34–13.80)	14.16 (13.41–14.91)
Intervention 1	*p =* 0.850	***p =* 0.014**	*p =* 0.734	*p =* 0.069	*p =* 0.271		*p =* 0.305	*p =* 0.514
Used	2.18 (1.73–2.72)	**8.39 (7.64–9.14)**	12.82 (12.08–13.56)	8.70 (8.21–9.20)	5.67 (5.37–5.96)		13.40 (12.55–13.24)	14.49 (13.65–15.33)
Not used	2.21 (1.76–2.66)	**7.77 (7.14–8.39)**	12.90 (12.29–13.52)	8.40 (7.99–8.81)	5.57 (5.31–5.80)		13.10 (12.40–13.81)	14.53 (13.85–15.21)
Intervention 2	*p =* 0.082	***p<*0.001**	*p =* 0.433	***p =* 0.034**	*p =* 0.121		*p =* 0.181	***p =* 0.041**
Used	2.37 (1.88–2.85)	**8.40 (7.73–9.06)**	12.99 (12.33–13.65)	**8.62 (8.18–9.06)**	5.65 (5.39–5.91)		13.39 (12.59–14.09)	**14.55 (13.78–15.31)**
Not used	2.11 (1.65–2.57)	**7.54 (6.91–8.18)**	12.83 (12.20–13.47)	**8.34 (7.92–8.76)**	5.52 (5.27–5.77		13.03 (12.32–13.75)	**14.06 (13.32–14.79)**

Linear Regression Model included gender, age group, skintype, region, education, self-reported weather, UVI and exposure scale. P-values are for test between groups and mean intervention levels.

* 95 confidence interval in parentheses

## Discussion

We showed a high fraction of usage of interventions, but no significant differences in pre-defined outcomes between the intervention groups; however, this may be caused by incomplete uptake of the interventions as we did find significant differences between users and non-users of the interventions. Users of the interventions improved the targeted behaviors, intervention 1 users improved avoidance e.g. shorter outdoor time between 11 am and 3pm, increased use of shade and staying inside and intervention 2 users improved sunscreen body coverage, fraction of time sunscreen used and use of wide brimmed hat. Usage of intervention 1 or 2 each improved the overall protection scale and importance to protect when going abroad. Users of intervention 2 had increased risk of sunburn. This could be caused by a higher awareness towards sunburn registration by participants who received a more comprehensive material than controls. Another explanation could be that a higher level of whole body sunscreen use instead of sunscreen use supplementing clothing, as this type of sunscreen use may have pit falls **[14]**. We did not find improved behaviors of receiving both interventions.

### Strength and limitations

The design of this study is the main strength together with a uv-dosimeter-validated questionnaire being able to adjust for exposure. The final sample was smaller than anticipated which could be due to a summer in 2018 in Denmark with more sun hours than average, which may have prompted more people to have their vacation in Denmark instead of going abroad. In addition, the uptake of interventions were smaller than expected. Inconsequent or insufficient usage of interventions could result in false security and sunburn and protection usage was not validated. Overall, the study had less power in regards to the pre-project goals **[32]**. Another limitation of the study is the lack of a baseline questionnaire due to the intermittent nature of a vacation and as such our groups could theoretically have had different baseline protection use despite randomization. Sunburn was self-reported and participants with more comprehensive interventions may have been more or less aware of sunburn or susceptible to report sunburn. The randomization procedure ensures similar groups; however, the study population could be more or less likely to consist of sun seekers compared to the background population. This will not influence results of the trial because they should be equally distributed across the study arms. However, it could limit generalizability and influence future implementation of the interventions. Finally, our study had an underrepresentation of men and lasting behavioral changes was not examined.

### Perspectives

Previously Buller et al. showed no improvements in behavior between pre-and post-measurement of a multifaceted intervention in North American resorts **[43]**. Another study showed single messages might be a good approach to communicate sun protection, which could explain the lack of dose-response in our study **[44]**. Our results are in line with previous result of the Campaign 2007–15 **[10, 45, 46]**. We have now developed methods, feasible to implement for persons travelling to destinations with high UVI. The level of usage of the interventions could be improved in future studies, however sun protection behavior is one motivation among a long list of other motivations (activities, behaviors, possibilities), when people travel. Feasibility, ease of application and lack of conflict with other vacation motivations could be focused on.

### Expected influence on future research in skin cancer prevention

The efficacy of sun protection methods is central to skin cancer research. Ninety percent of skin cancers could be prevented by a reduction in UVR exposure **[4]**. One week of sunny vacation was estimated to expose an individual to more than 40% of the annual dose received in Denmark **[22]**. Methods to reduce UV-exposure to the skin are well-known, however, the evidence of current prevention methods to reduce individual UVR exposure in practice is insufficient. Thus, it is important to establish evidence of current sun protection methods and to develop new methods to improve them where current methods are inadequate as skin cancer rates are high compared to how easily they could be decreased. Providing hats for people may not be possible on a population level; however, it was possible to increase hat use by increasing availability in Denmark. The level of sun protection from sunscreen use can be improved in several ways: number of application times, applied amount and coverage fraction of the intended skin area to protect. Receiving information on how to schedule activities exposed vs less exposed respectively before/after vs during the interval 11am-3pm makes it possible to avoid more than 50% of the 24h total ambient radiation. Dissemination of the UVI alone previously didn’t influence people’s behavior **[47];** however, it has been able to increase awareness **[48],** which is a precursor to changed behavior in behavioral models **[33]** but we used it as part of our intervention which actually showed an influence on the behavior. Additionally, sun safety smartphone applications have shown indications of improvement in sun protection practices in a randomized trial **[49]**. Furthermore, we showed an increased risk of sunburn for travelers, with increasing UVI of 1.22/UVI level of the destination, which increases the relevance of using this information for the users i.e. when going to e.g. northern Spain a destination with a UVI three levels higher compared to Denmark the risk of sunburn increases to 1.82, while at e.g. canary islands, Spain, five UVI levels higher increases this risk to 2.70.

Our intervention messages will be added to future campaigns as a tool to reach the Danes when dispersed all over the world and thereby reduce the UVR exposure on a population level and eventually prevent skin cancers in Denmark. In addition, reductions in UVR exposure of the Danes will decrease the economic costs of skin cancer **[50**–**52]**. Increasing the uptake of the interventions is likely to increase the population protection behavior and thereby reducing the exposure and in a long time perspective the skin cancer rates and as such improve the return on investment.

## Conclusion

Simple measures can help avoid the majority of the most widespread cancer worldwide. Thoughtfulness planning your activities and thoroughness in applying sunscreen are keywords in terms of behavior and protection in the sun. Sunny vacations has a major influence on the annual Danish UV-exposure and here we show that it is possible to influence travelers to protect themselves better and to increase sun protection behavior.

## Supporting information

S1 Checklist (DOC)

S1 Material Intervention materials. (PDF)

S1 File (DOCX)

S2 File (PDF)

S3 File (PDF)
