# Supplementary material for: Novel sunprotection interventions to prevent skin cancer: A randomized study targeting Danes going on vacation to destinations with high UV index
Source: PLoS One. 2020 Dec 31;15(12):e0244597. doi: 10.1371/journal.pone.0244597 (PMC7774952; doi:10.1371/journal.pone.0244597)
Supplement: S1 Material — (PDF) [file pone.0244597.s002.pdf]

Aktivitetsplanlægning

Solens uv-stråling er stærkest mellem kl. 12 og 15, og ca. halvdelen af dagens uv-stråling falder i dette tidsrum. Desuden er uv-strålingen midt på dagen stærkere i lande tættere på ækvator end i Danmark. Du kan derfor undgå meget af solens uv-stråling på din ferie ved at finde skygge midt på dagen.

Skal du opholde dig i direkte sol, så gør det så vidt muligt før kl. 12 og/eller efter kl. 15, da du således mindsker risikoen for at blive solskoldet på din ferie. Uanset tidspunkt skal du stadig huske at beskytte dig, når uv-indekset er over 3.

På bagsiden er eksempler på nogle af de aktiviteter, som danskerne ofte foretager sig og som de har vurderet ift. skygge. Du kan med fordel bytte om på aktiviteter i løbet af dagen, så du i højere grad opholder dig i skyggen midt på dagen.

Ikke alle aktiviteter er enten sol- eller skyggeaktiviteter, men **vi vil gerne bede dig overveje dine egne aktiviteter. Kan de planlægges så du undgår den stærke sol midt på dagen?**

Du kan også få nogle fif på **youtube. Søg på *Help a Dane* kampagne**. Her giver de lokale gode råd til solbeskyttende aktiviteter på nogle af danskernes foretrukne feriedestinationer.

Aktiviteter på ferien

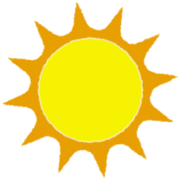

Solaktiviteter

Aktiviteter som danskerne primært foretager sig mellem kl. 12-15.

Vi anbefaler, at du foretager disse **før kl. 12 og/eller efter kl. 15:**

- Bade i poolen/havet
- Byvandring
- Bådtur
- Cykle
- Delfin- og hvalsafari
- Gåtur
- Køre på scooter
- Naturoplevelser
- Sejle på jetski/vandscooter
- Sightseeing
- Snorkle
- SUP/Surfe
- Vandland
- Vandretur
- Vandsport

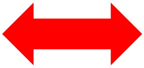

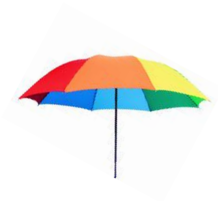

Skyggeaktiviteter

Aktiviteter som danskerne primært foretager sig før kl. 12 og/eller efter kl. 15.

Vi anbefaler, at du i højere grad foretager disse **mellem kl. 12-15:**

- Dykning
- Læse i skyggen/indendørs
- Massage
- Meditation
- Sove
- Spille spil i skyggen/indendørs
- Café- og restaurantbesøg
- Shopping

Kære Deltager

Tusind tak fordi du vil deltage i vores undersøgelse af forskellige solbeskyttelsesmetoder, når danskerne er på solferie.

Vi har sendt dette brev både som mail og vedlagt det i det tilsendte materiale. Det er **vigtigt at du læser hele materialet** nøje igennem.

I materialet finder du følgende information om solbeskyttelse, vi vil opfordre dig til at følge på din solferie:

Søg skygge mellem kl. 12 og 15

Kort fortalt går denne form for solbeskyttelse ud på at undgå den stærke middagssol, og dermed mindske din risiko for solskoldninger på din ferie. **Du bedes gøre følgende**:

1. Download og anvend Solkampagnens **Uv-indeks-app**, som kan vise uv-indekset på din feriedestination. Gør det gerne forud for din ferie, hvis du ikke har internetdækning mens du er afsted.
2. Prøv vores **Hudtypeguide** og se, hvor meget sol netop din hud kan tåle.
3. Planlæg din dag med inspiration fra vores **Aktivitetsplanlægger**, og minimer din tid i direkte sol.

Du finder yderligere information til hver af delelementerne i det vedhæftede/vedlagte materiale.

Efter ferien vil du få tilsendt et **spørgeskema** fra os, som vi håber du vil besvare. Det er vigtigt at vi får din besvarelse, uanset hvor meget du har fulgt anvisningerne om solbeskyttelse.

Har du nogen spørgsmål til din deltagelse i testen, så kan du kontakte os på:

brk@cancer.dk eller 3525 7666

Vi håber du får en rigtig god ferie ☺

Venlig hilsen

Brian Køster

Forebyggelse og Oplysning

Kræftens Bekæmpelse

Solbeskyttelse på solferie

Når du rejser sydpå til lande, der er tættere på ækvator med et varmere klima end i Danmark, er det vigtigt at huske solbeskyttelse. Solens uv-stråling er nemlig stærkere, jo tættere du kommer på ækvator.

Du skal derfor være opmærksom på, at der går kortere tid før du bliver solskoldet af solen, hvis du ikke solbeskytter dig, end hvis du var i Danmark.

Solbeskyttelse – og især på solferier – er vigtigt, da solskader øger risikoen for at udvikle hud- og modermærkekræft senere i livet.

I Danmark bliver der hvert år konstateret ca. 16.000 nye tilfælde af hud- og modermærkekræft, og kræftformerne er dermed de hyppigst forekommende blandt danskerne.

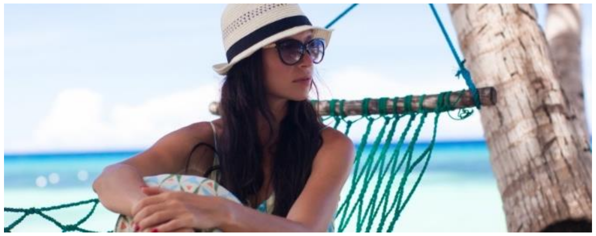

Uv-indekset på solferier

Uv-indekset er et udtryk for intensiteten af solens ultraviolette stråling, og dermed hvornår man skal passe på i solen.
Når uv-indekset er 3 eller mere, bør du beskytte dig selv og dine kære mod uv-stråling. Det høje uv-indeks kombineret med at man på solferie oftest er mere udendørs end vanligt - og med mindre tøj på - gør det særligt vigtigt at beskytte sig mod solens uv-stråling.

Skru ned for solen

Du kan let forebygge solskoldninger samt hud- og modermærkekræft ved at beskytte dig i sommersolen. Vi anbefaler, at du beskytter dig mod solens uv-stråling ved brug af solrådene:,
skygge, tøj, solhat og solcreme
når uv-indekset er 3 eller højere - <u>også</u> *uden for* tidsrummet mellem kl. 12 & 15.

Læs mere om forebyggelse af kræft i huden på:
www.solkampagnen.dk

SKRU NED FOR SOLEN MELLEM 12 & 15

Kræftens Bekæmpelse

TrygFonden

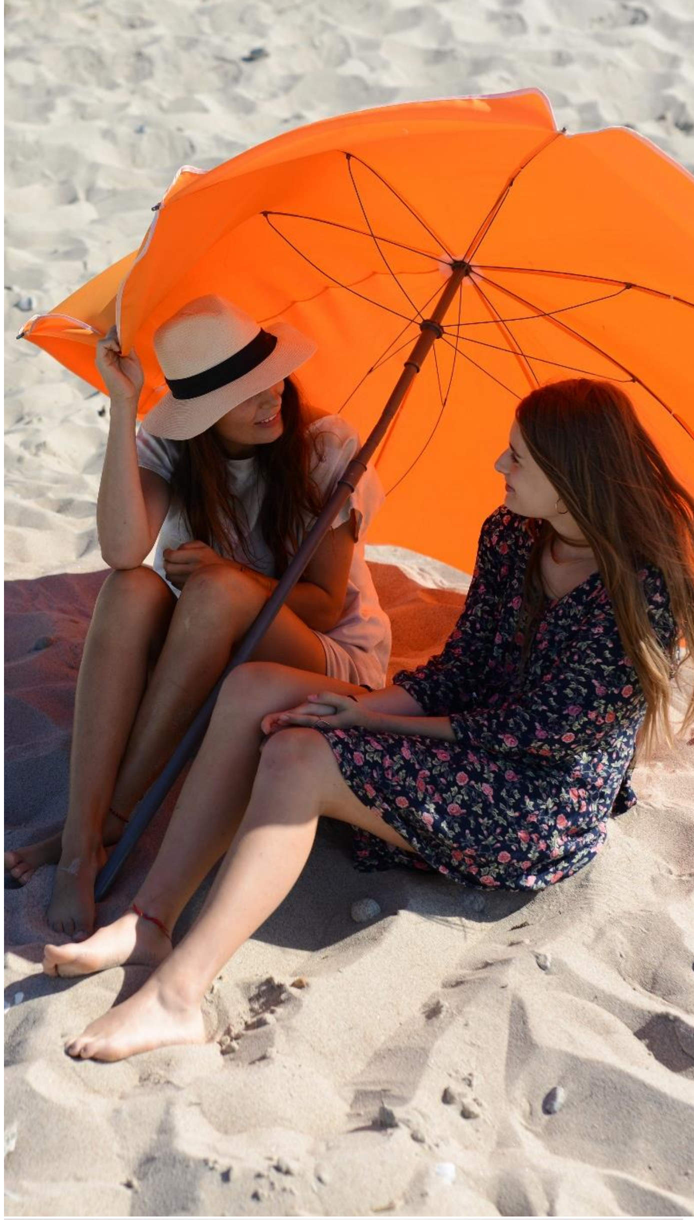

DIN GUIDE TIL SOLBESKYTTELSE

- Skygge

Skygge

Søg ind i skyggen mellem kl. 12 & 15, når uv-indekset er 3 eller højere

Solens uv-stråling er stærkest mellem kl. 12 & 15. Halvdelen af dagens uv-stråling falder i dette tidsrum. Du kan derfor undgå meget af solens uv-stråling på din ferie ved at finde skygge midt på dagen. Tænk over det, når dagens aktiviteter skal planlægges. Skal du opholde dig i direkte sol, så gør det så vidt muligt før kl. 12 og/eller efter kl. 15.

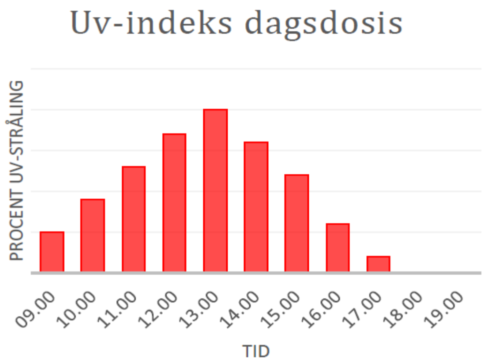

Figuren viser et eksempel på fordeling af dagens uv-strålingsdosis. Fordelingen kan variere mellem destinationer, men er typisk højest omkring 13 (12-14 - afhængig af tidszone). Find din destination i appen

I vedlagte aktivitetsplanlægning kan du inden afrejse og undervejs på ferien finde inspiration til *Skyggeaktiviteter*, som anbefales mellem kl. 12-15, og *Solaktiviteter*, der anbefales før kl. 12 og efter kl. 15.

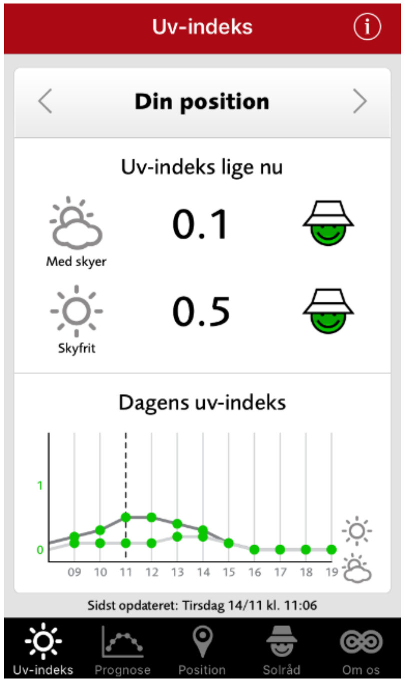

Uv-indeks-appen

I appen 'Uv-indeks' kan du få dagens forventede uv-indeks for både ind- og udland. Du kan også se en femdagsprognose og få gode råd til solbeskyttelse. Derudover kan appen hjælpe dig under forberedelserne til rejsen, da du nemt kan tjekke op på, hvilken form for solbeskyttelse, du skal bruge på ferien, og hvilken faktor solcreme, der er fornuftig på dit feriested.

Instruktion til appen

Vi vil bede dig downloade appen 'Uv-indeks' på din smartphone eller tablet fra App Store eller Google Play før din ferie.

Sådan bruger du appen:

- Søg på 'Uv-indeks'
- Installér og giv tilladelse til anvendelse af *push-meddelelser* og *lokation*.

Appen har forskellige funktioner, vi gerne vil bede dig om at anvende:

- Tryk på 'Position' og indtast din feriedestination og tilføj denne som favorit.
- Tryk på 'Rediger favoritter og advarsler' og vælg *Advarselsfunktionen* 'Hver dag' for at få en daglig notifikation om dagens højeste uv-indeks på din lokation.
- Før din ferie kan du trykke på 'Prognose' og se hvor højt uv-indekset er på din feriedestination.
- Tryk på 'Solråd'. Her kan du læse om de *Generelle solråd*
- Tag **hudtypeguiden** og se hvor længe din hud kan tåle at være ude i solen uden solbeskyttelse.

PRØV HUDTYPEGUIDEN

Svar på de seks spørgsmål og læg pointene sammen.
Så får du et bud på din hudtype og den nødvendige solbeskyttelse.

1 Hold paletten med de syv nuancer mod undersiden af din underarm, hvor din hud typisk ikke er så eksponeret for solens stråling. Hvad er din hudnuance?

- ◯ A ..... **0 p.**
- ◯ B ..... **0 p.**
- ◯ C ..... **1 p.**
- ◯ D ..... **1 p.**
- ◯ E ..... **2 p.**
- ◯ F ..... **2 p.**
- ◯ G ..... **3 p.**

2 Bliver du skoldet i den første sommersol, hvis du ikke beskytter dig?

- ◯ Bliver altid skoldet ..... **0 p.**
- ◯ Bliver sommetider skoldet ..... **1 p.**
- ◯ Bliver sjældent skoldet ..... **2 p.**
- ◯ Bliver aldrig skoldet ..... **3 p.**

3 Bliver du brun i den første sommersol, hvis du ikke beskytter dig?

- ◯ Bliver aldrig brun ..... **0 p.**
- ◯ Bliver lidt brun ..... **1 p.**
- ◯ Bliver noget brun ..... **2 p.**
- ◯ Bliver kraftig brun ..... **3 p.**

4 Hvad er din øjenfarve?

- ◯ Blå ..... **0 p.**
- ◯ Grå ..... **1 p.**
- ◯ Grøn ..... **1 p.**
- ◯ Brun ..... **2 p.**

5 Hvad er (var) din naturlige hårfarve, da du var 18 år?

- ◯ Rødt ..... **0 p.**
- ◯ Blond ..... **0 p.**
- ◯ Lysebrunt ..... **1 p.**
- ◯ Mørkebrunt ..... **2 p.**
- ◯ Sort ..... **2 p.**

6 Har du fregner?

- ◯ Ja, mange ..... **0 p.**
- ◯ Ja, få ..... **1 p.**
- ◯ Nej ..... **2 p.**

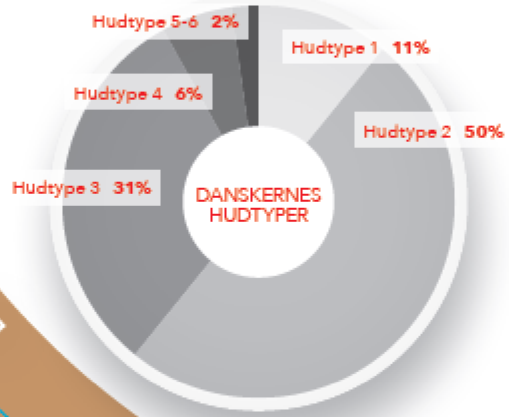

Tæl dine point sammen og skriv tallet her: ◯

Ud fra dine svar har du følgende hudtype:

Hudtype 1	**0-3 p.**
Hudtype 2	**4-7 p.**
Hudtype 3	**8-11 p.**
Hudtype 4	**12-14 p.**
Hudtype 5-6	**15 p.**

G A B C E F

LÆS OM DIN HUDTYPE

Uanset hudtype er solbeskyttelse vigtig. Brug de tre solråd: Skygge, solhat/tøj og solcreme. Ved et uv-indeks på 3 eller derover anbefaler vi, at du solbeskytter dig. Du kan følge uv-indekset i vores app UV-INDEKS. Vi anbefaler også, at du holder øje med din hud og kontakter din læge, hvis du oplever hudforandringer.
Se mere på skrunedforsolen.dk

HUDTYPE 1

Du har en sart hud, der let får for meget sol. Du bliver let solskoldet, og din hud bliver sjældent/aldrig solbrun. Solbeskyttelse er næsten altid nødvendigt, når du er udenfor om sommeren i Danmark og på solferie i udlandet.

Uden solbeskyttelse risikerer du solskoldning, hvis du er ude mere end:
- Ved uv-indeks 3 .... **40 min.**
- Ved uv-indeks 7 .... **15 min.**
- Ved uv-indeks 11 .... **10 min.**

HUDTYPE 2

Du har en sart hud, der kan tåle lidt mere sol end hudtype 1, men du bør stadig være forsigtig, når du er udenfor, og uv-indekset er 3 eller mere. Din hud bliver let solskoldet, men du kan godt blive solbrun.

Uden solbeskyttelse risikerer du solskoldning, hvis du er ude mere end:
- Ved uv-indeks 3 .... **55 min.**
- Ved uv-indeks 7 .... **20 min.**
- Ved uv-indeks 11 .... **15 min.**

HUDTYPE 3

Hudtype 3 er mindre sart end 1 og 2. Du bliver let solbrun, men kan også blive forbrændt, hvis du ikke passer på i solen. Husk, at det aldrig er sundt at stege i solen. Heller ikke, selvom du ikke solskoldes.

Uden solbeskyttelse risikerer du solskoldning, hvis du er ude mere end:
- Ved uv-indeks 3 .... **65 min.**
- Ved uv-indeks 7 .... **25 min.**
- Ved uv-indeks 11 .... **15 min.**

HUDTYPE 4

Hudtype 4 kan tåle en del sol, men husk, at det aldrig er sundt at stege i solen. Heller ikke, selvom man ikke solskoldes. Livstidseksponeringen for uv-stråling har også betydning for risikoen for hud- og modermærkekræft.

Uden solbeskyttelse risikerer du solskoldning, hvis du er ude mere end:
- Ved uv-indeks 3 .... **95 min.**
- Ved uv-indeks 7 .... **40 min.**
- Ved uv-indeks 11 .... **25 min.**

HUDTYPE 5-6

Hudtype 5 og 6 kan tåle meget sol, men det er vigtigt at huske, at det aldrig er sundt at stege i solen. Heller ikke, selvom man ikke solskoldes.

Uden solbeskyttelse risikerer du solskoldning, hvis du er ude mere end:
- Ved uv-indeks 3 .... **130 min.**
- Ved uv-indeks 7 .... **55 min.**
- Ved uv-indeks 11 .... **35 min.**

HUDTYPE-GUIDEN ER KUN VEJLEDENDE

Husk din sunde fornuft i solen, og vær altid opmærksom på din hud.

Varenummer 5229. Maj 2016

Kære Deltager

Tusind tak fordi du vil deltage i vores test af forskellige solbeskyttelsesmetoder, når danskerne er på solferie.
Vi har sendt dette brev både som mail og vedlagt det i det tilsendte materiale. Det er **vigtigt at du læser hele materialet** nøje igennem.
I materialet finder du følgende information om solbeskyttelse, vi vil opfordre dig til at følge på din solferie:

Husk Solhatten

Brug masser af Solcreme

Kort fortalt går denne form for solbeskyttelse ud på at beskytte kroppens udsatte områder mod solens stråling ved både at bruge en solhat og masser af solcreme, således at du mindsker din risiko for solskoldninger på din ferie. **Du bedes gøre følgende**:

1. Brug en **bredskygget solhat,** når du er direkte i solen – og især mellem kl. 12 og 15.
2. Følg vores nemme **indsmøringsguide**, når du skal smøre dig ind i **solcreme**.

Du finder yderligere information til hver af delelementerne i det vedhæftede/vedlagte materiale.

Efter ferien vil du få tilsendt et **spørgeskema** fra os, som vi håber du vil besvare. Det er vigtigt at vi får din besvarelse, uanset hvor meget du har fulgt anvisningerne om solbeskyttelse.

Har du nogen spørgsmål til din deltagelse i testen, så kan du kontakte os på:
brk@cancer.dk eller 3525 7666

Vi håber du får en rigtig god ferie ☺

Venlig hilsen

Brian Køster
Forebyggelse og Oplysning
Kræftens Bekæmpelse

Solbeskyttelse på solferie

Når du rejser sydpå til lande, der er tættere på ækvator med et varmere klima end i Danmark, er det vigtigt at huske solbeskyttelse. Solens uv-stråling er nemlig stærkere, jo tættere du kommer på ækvator.

Du skal derfor være opmærksom på, at der går kortere tid før du bliver solskoldet af solen, hvis du ikke solbeskytter dig, end hvis du var i Danmark.

Solbeskyttelse – og især på solferier – er vigtigt, da solskader øger risikoen for at udvikle hud- og modermærkekræft senere i livet.

I Danmark bliver der hvert år konstateret ca. 16.000 nye tilfælde af hud- og modermærkekræft, og kræftformerne er dermed de hyppigst forekommende blandt danskerne.

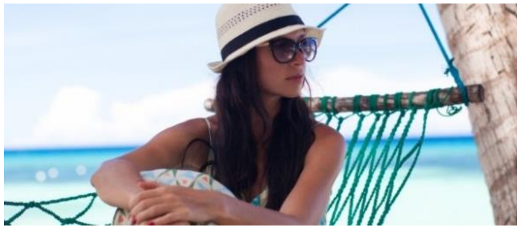

Uv-indekset på solferier
Uv-indekset er et udtryk for intensiteten af solens ultraviolette stråling, og dermed hvornår man skal passe på i solen.

Når uv-indekset er 3 eller mere, bør du beskytte dig selv og dine kære mod uv-stråling.

Det høje uv-indeks kombineret med at man på solferie oftest er mere udendørs end vanligt - og med mindre tøj på, gør det særligt vigtigt at beskytte sig mod solens uv-stråling.

Skru ned for solen
Du kan let forebygge solskoldninger samt hud- og modermærkekræft ved at beskytte dig i sommersolen. Vi anbefaler, at du beskytter dig mod solens uv-stråling ved brug af solrådene:

Skygge, tøj, solhat og solcreme
når uv-indekset er 3 eller højere - <u>også</u> *uden for* tidsrummet mellem kl. 12 og 15:

SKRU NED FOR SOLEN MELLEM 12 & 15

Kræftens Bekæmpelse

TrygFonden

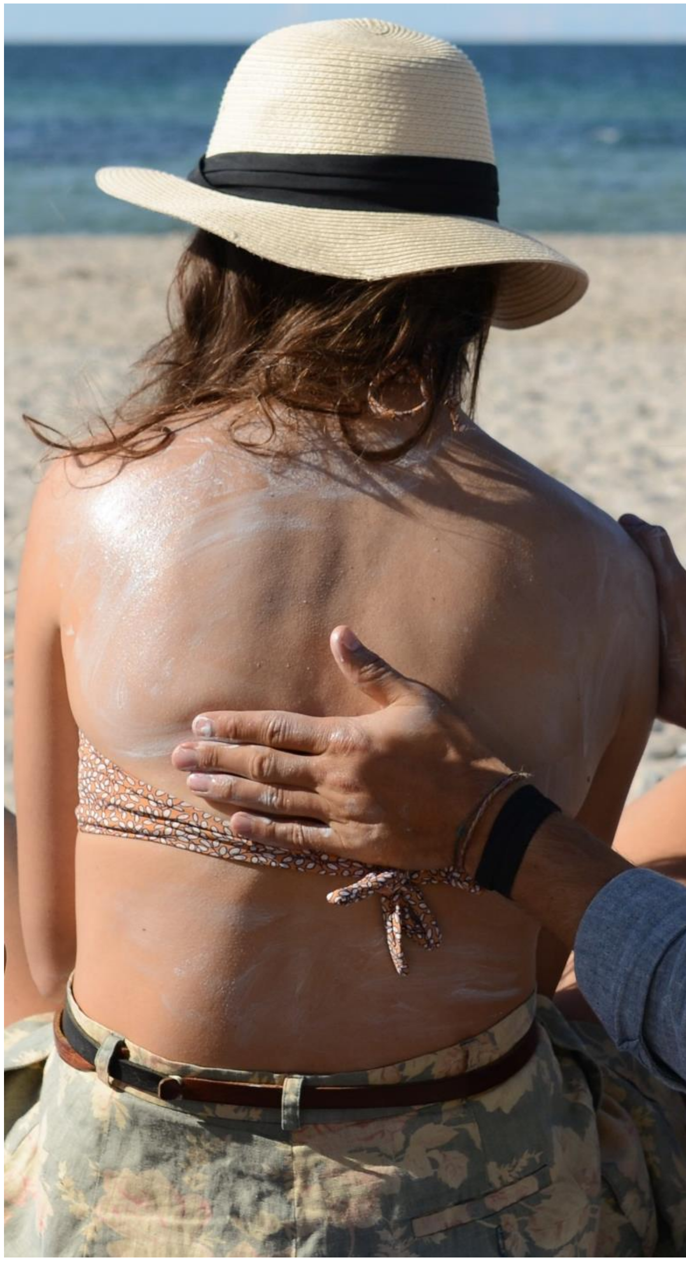

DIN GUIDE TIL SOLBESKYTTELSE

- Solhat og solcreme

Solhat

Medbring altid din egen mulighed for skygge på solferien

Hvis du er direkte ude i solen - og især mellem kl. 12 og 15 - og ikke har mulighed for at være i skyggen, så er en solhat med bred skygge den bedste måde at solbeskytte hoved- og halsområdet mod solskoldning.

3 ud af 4 tilfælde af almindelig hudkræft i Danmark forekommer i hoved- og halsregionen, og områderne udgør derfor kroppens mest udsatte områder, når man er ude i solen.

Fakta om solhatten:

- På din solferie vil vi bede dig om, at bruge **en bredskygget solhat** når uv-indekset er 3 eller højere - og især mellem kl. 12 og 15.
- Har du ikke selv en bredskygget solhat til rådighed, kan du bruge den tilsendte hat.
- Den er i et sammenklappeligt design, som gør det nemt at tage med i tasken, når du skal ud i solen.
- Vi anbefaler *ikke* brugen af kasketter som solbeskyttelse, da de ikke beskytter ører, nakke og hals tilstrækkeligt.

Solcreme

Husk altid at bruge masser af solcreme der, hvor skygge eller tøj ikke dækker

Rigtig mange danskere bliver desværre solskoldede, selvom de bruger solcreme. Det kan skyldes forkert indsmøringsteknik eller påsmøring af et for tyndt lag solcreme. Dette medvirker, at **beskyttelsen falder markant fx bliver faktor 15 til faktor 3, hvis kun halvdelen af den anbefalede mængde påsmøres**.

For at gøre indsmøringen lidt nemmere, kan du med fordel anvende den vedlagte guide til at huske solcreme på hele kroppen:

Ansigt – Hals – Nakke – Bryst –
Arme – Skuldre – Mave – Ryg – Ben.

Husk at **smøre alle kroppens områder grundigt** ind med solcreme – et område ad gangen inden du tager tøj på og går ud. **Gennemsnitligt glemmer vi 1/5 af kroppen, når vi smører os ind i solcreme.** Vær opmærksom på at få hjælp, hvis der er områder du ikke selv kan nå.

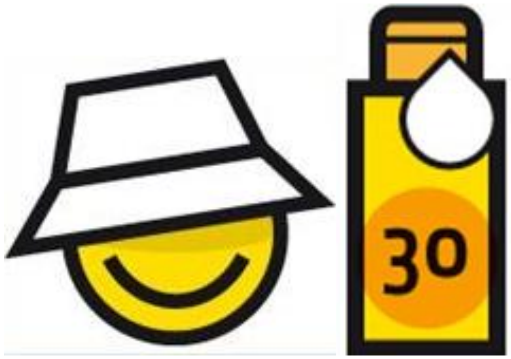

Vidste du, at…

-Du typisk har brug for solcreme med **faktor 30-50**, når du skal på solferie.

-Du kan opnå bedre beskyttelse med solcreme ved at **smøre dig ind to gange**: mindst 20 minutter før du skal ud i solen, og igen umiddelbart inden du går ud.

-Du bør **smøre dig ind i solcreme igen**, hvis du har været i vandet eller har svedt

-Du bør huske at **genindsmøre** dig hver 2.-3. time for at bevare beskyttelsesvirkningen.

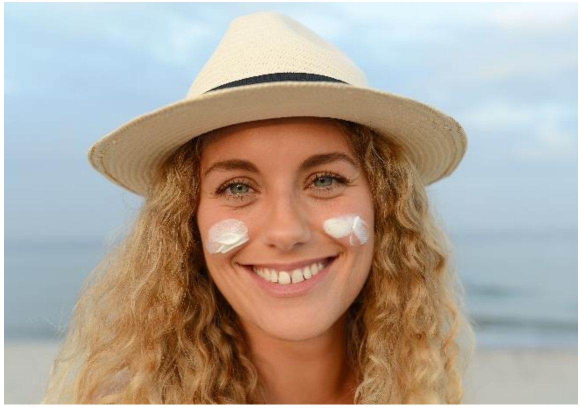

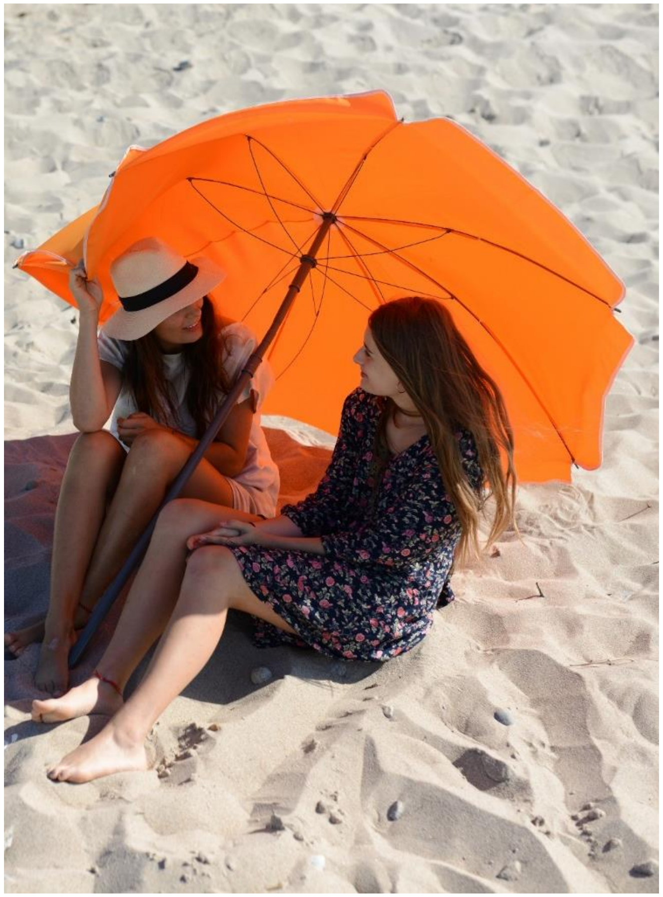

DIN GUIDE TIL SOLBESKYTTELSE

- Solferie

Solbeskyttelse på solferie

Når du rejser sydpå til lande, der er tættere på ækvator med et varmere klima end i Danmark, er det vigtigt at huske solbeskyttelse. Solens uv-stråling er nemlig stærkere, jo tættere du kommer på ækvator.

Du skal derfor være opmærksom på, at der går kortere tid før du bliver solskoldet af solen, hvis du ikke solbeskytter dig, end hvis du var i Danmark.

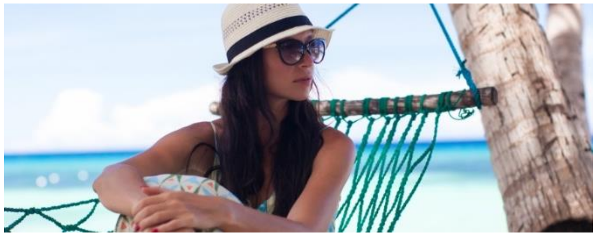

SKRU NED FOR SOLEN MELLEM 12 & 15

Uv-indekset på solferier

Uv-indekset er et udtryk for intensiteten af solens ultraviolette stråling, og dermed hvornår man skal passe på i solen.

Når uv-indekset er 3 eller mere, bør du beskytte dig selv og dine kære mod uv-stråling.

Det høje uv-indeks kombineret med, at man på solferie oftest er mere udendørs end vanligt - og med mindre tøj på - gør det særligt vigtigt at beskytte sig mod solens uv-stråling.

Skru ned for solen

Du kan let forebygge solskoldninger samt hud- og modermærkekræft ved at beskytte dig i sommersolen. Vi anbefaler, at du beskytter dig mod solens uv-stråling ved brug af solrådene:

Skygge, tøj, solhat og solcreme

, når uv-indekset er 3 eller højere.

Kræftens Bekæmpelse

TrygFonden

Kære Deltager

Tusind tak fordi du vil deltage i vores undersøgelse af anvendeligheden af solbeskyttelsesrutiner, når danskerne er på solferie.

Vi har sendt dette brev både som mail og vedlagt det i det tilsendte materiale. Det er **vigtigt at du læser hele materialet** nøje igennem.

I materialet finder du følgende information om solbeskyttelse, vi vil opfordre dig til at følge på din solferie:

Søg Skygge mellem kl. 12 og 15

Brug masser af Solcreme

Husk Solhatten

Kort fortalt går din solbeskyttelse ud på at undgå den stærke middagssol og beskytte kroppens udsatte områder mod solens stråling ved at bruge en solhat og masser af solcreme, således at du mindsker din risiko for solskoldninger på din ferie. **Du bedes gøre følgende:**

1. Download og anvend Solkampagnens **Uv-indeks-app**, som kan vise uv-indekset på din feriedestination.
2. Prøv vores **Hudtypeguide** og se, hvor meget sol netop din hud kan tåle.
3. Planlæg din dag ved hjælp af vores **Aktivitetsplanlægger**, og minimer din tid direkte i solen.
4. Brug en **bredskygget solhat,** når du er direkte i solen – og især mellem kl. 12 og 15.
5. Følg vores nemme **indsmøringsguide**, når du skal smøre dig ind i **solcreme**.

Du finder yderligere information til hver af delelementerne i det vedhæftede/vedlagte materiale.

Efter ferien vil du få tilsendt et **spørgeskema** fra os, som vi håber du vil besvare. Det er vigtigt at vi får din besvarelse, uanset hvor meget du har fulgt anvisningerne om solbeskyttelse.

Har du nogen spørgsmål til din deltagelse i testen, så kan du kontakte os på:

brk@cancer.dk eller 3525 7666

Vi håber du får en rigtig god ferie ☺

Venlig hilsen

Brian Køster

Forebyggelse og Oplysning

Kræftens Bekæmpelse

Kræftens Bekæmpelse

Kære Deltager

Tusind tak fordi du vil deltage i vores undersøgelse af anvendeligheden af solbeskyttelse, når danskerne er på solferie.

Vi har sendt dette brev både som mail og postalt. Din deltagelse i det videnskabelige projekt er meget værdifuld for os der arbejder med at forebygge kræft i huden. Ved at deltage i projektet **bidrager du til at flere danskere kan undgå at få hudkræft.**

Den solbeskyttelse vi vil bede dig følge er solkampagnens solråd, således at du mindsker din risiko for solskoldninger på din ferie. Vi vil bede dig huske følgende på din ferie:

Søg Skygge

Brug Solhat/Tøj

Brug Solcreme

Derudover skal du blot nyde ferien. **Efter ferien** vil du få tilsendt et **spørgeskema** fra os, som du bedes besvare.

Det er vigtigt at vi får din besvarelse, uanset hvor meget du har fulgt anvisningerne om solbeskyttelse.

Har du nogen spørgsmål til din deltagelse i undersøgelsen, så kan du kontakte os på:

brk@cancer.dk eller 3525 7666

Vi håber du får en rigtig god ferie ☺

Venlig hilsen

Brian Køster

Forebyggelse og Oplysning

Kræftens Bekæmpelse

Kræftens Bekæmpelse

	Group 1	Group 2	Group 3	Group 4
**Intervention 1**	**X**		**X**	
Shade and sun avoidance information	X		X	
Skin type test	X		X	
UV-indeks app	X		X	
Activity planner	X		X	
**Intervention 2**		**X**	**X**	
Sunscreen and hat Information		X	X	
Detailed sunscreen application guide		X	X	
Wide Shade Hat		X	X	
**Minimal intervention**				X
Use sun advice; Shade, Hat, Sunscreen				X
