# Supplementary material for: Novel sunprotection interventions to prevent skin cancer: A randomized study targeting Danes going on vacation to destinations with high UV index
Source: PLoS One. 2020 Dec 31;15(12):e0244597. doi: 10.1371/journal.pone.0244597 (PMC7774952; doi:10.1371/journal.pone.0244597)
Supplement: S1 File — (DOCX) [file pone.0244597.s003.docx]

**Test of developed interventions to prevent skin cancer: a randomized study targeting Danes going on vacation to sunny destinations with high UV index**

**Aim**

The aim of the study is to test a series of developed effective interventions targeting Danes going on vacation to sunny destinations to decrease sunburn by increasing use of shade, hats, protective clothing, and sunscreen to prevent skin cancer in the Danish population.

**Prevalence of skin cancer and sun exposure**

Incidence of malignant and non-malignant skin cancer have increased for decades in large parts of the world and especially in Caucasian populations **[1]**. In Denmark, almost 4.000 individuals are diagnosed with a skin cancer annually (basal cell cancer not included) and more than 300 die from a skin cancer **[2]**. Exposure to ultraviolet radiation (UVR) from the sun and from artificial sources e.g. sunbeds is the main risk factor for skin cancer **[3]**. Sunburn is both a measure of the dose of UVR exposure received as well as an independent risk factor for skin cancer **[4]**. It has been estimated that up to 90 % of all skin cancers could be avoided by behavioral changes **[5, 6]**. One of the main sources of UVR exposure in the Danish population is vacations to destinations with high UV index (UVI) **[7-11]**.

**The Danish sun safety campaign**

In 2007, the Danish Sun Safety Campaign was launched in a partnership between the Danish Cancer Society and the Danish foundation, TrygFonden. The purpose of the campaign is to prevent skin cancer in Denmark by increasing the use of appropriate sun protection in the natural sun and avoiding sunbed use in the Danish population. This study is going to be part of the Danish Sun Safety Campaign. Thus, experience from the campaign will be implemented in the study and study results will be used in the campaign afterwards.

The Danish Sun Safety Campaign’s sun advice to prevent exposure to UVR from the natural sun are structured in three prioritized messages:

*Shade* is the primary message, which embraces not only staying in the shade, but also staying out of the sun when the UVI reaches daily maximum (between 12 and 3 pm). The secondary message is *hat and ‘shade for the body’* meaning clothes. The tertiary message is *sunscreen*. The messages should be applied in combination to be effective.

The priority of the messages are in accordance with the European code against cancers suggestions for individual’s protection against UVR. Avoidance of UVR by staying indoor in the middle of the day when UVR levels are highest obviously provides the best protection. Shade structures provide low protection against UVR (SPF = 2-5), due to reflection and diffuse UVR **[12]**. Textiles, like clothing and hats provides high protection of the covered areas (SPF > 50)**[13]**, while hats only provide low protection on shaded areas, like the face (SPF 2-4)**[14]**. Sunscreens are produced with a theoretical SPF up to 50 in Denmark (requires distribution of 2 mg/ml) which is categorized as high. Consumers only apply approximately 0.5-1.0 mg/ml sunscreen, which means that the actual protection of sunscreens labeled with SPF 16 and SPF 36 drops below SPF 4 and SPF 6, respectively, which corresponds to low protection **[15]**.

The campaign has achieved substantial successful results:

- The fraction of sunburn in the population in the Danish summer decreased from 44 % in 2008 to 22 % in 2014 **[16]**
- Use of sunbed decreased from 26 % in 2007 to 9 % in 2014 **[17]**

However:

- The fraction of sunburn in the population going on vacations to sunny destinations with high UVI was largely unchanged from 25 % in 2008 to 27 % in 2014 **[18]**

**Vacations to sunny destinations is the most important arena for melanoma intervention**

Results from the large Norwegian Women’s Cohort (NOWAC) with 100.000+ women showed an OR of 1.7 (1.2-2.5) for MM when comparing one or more annual vacations to sunny destinations with less than one annual vacation. Each year, almost 50 % of the Danish population is going on a vacation to a sunny destination. Studies have shown **[19, 20]** that Danes going on vacation to destinations with high UVI receive 43 % of the annual UVR dose in just one week and found that all participants in the studies were sunburned (determined by objective measurement). Sunburn is generally underestimated by self-evaluation **[21]**. More than 90 % of the Danish population have skin type’s I-III. This means that the minimal erythemal dose (MED) will be exceeded by the vast majority of vacationers after only 20 minutes or less at UV 9 (e.g. in France and Italy) or 15 min or less at UV 11 (e.g. in Thailand and the Canary Islands) if not protected, which results in sunburn and increased risk of skin cancer. Vacations to sunny destinations generally is a skin cancer risk factor, especially for people of Caucasian origin **[22, 23]**.

**Challenges with implementation and adherence to the sun advice**

When Danes are travelling to sunny destinations, they bring their sun-related behavior from the north, which is not adapted to the high UVI of the destination. For instance, the Danes do not take a siesta, a break from the sun, like several local populations. During 11am-3pm, when UVI is highest, 90 % of Danes on vacation are outside more than an hour, 72 % more than 2 hours and 41 % are outside more than 3 hours**[24]**. This is problematic as the dose and intensity of the radiation during prolonged times outdoors during vacations, especially to locations at lower latitudes, increases risk of sunburns. In addition, Danes do not have a strong tradition of wearing hats. The hat advice has the lowest penetration in the population. About 80 % of the Danes never wore a hat during their vacation and only 10 % used it often or always **[24]**. This is very unfortunate because more than 75 % of non-melanoma skin cancers arise in the head, neck and scalp region **[25]**. Sunscreen is the sun protection advice with the highest penetration in the population. Sunscreen has reduces squamous cell skin cancer and melanoma under high UVR conditions in Australia **[26, 27]**; however the evidence for the protective effects of sunscreen against sunburn and melanoma is ambiguous **[28]**. Sunscreen with sun protection factor (SPF) 15 only lets 1/15 corresponding to ~ 7% of the UV radiation through. However, in real life there are many pitfalls when using sunscreen that can reduce its effectiveness, e.g. application of insufficient amount, lack of re-application, missing spots, use of insufficiently low SPF, application after going outdoors in the sun, as well as behaviors reversing the protective effect of sunscreen such as increased duration of sun exposure with sunscreen and use of sunscreen for intentional tanning **[10, 15, 29, 30]**. Studies have shown that the average sunscreen application of the Danes is less than 50 % of the recommended amount **[15, 31]**. In addition, more than 50 % of the Danes applying sunscreen used a sunscreen with insufficient sun protection factor (SPF < 30) on their vacation and only 25 % re-applied sunscreen sufficiently.

Therefore, there is a need to test and develop effective interventions that can improve these sun protection practices and reduce UVR exposure of Danes going on vacation to sunny destinations with a high UVI to prevent skin cancers in Denmark.

**Intervention study**

To achieve the aim of this study we will conduct a randomized controlled trial with three arms during May-Dec 2017. Participants will be Danish adults traveling on vacation in 2018 for a period of 1-3 weeks, recruited from the civil registration system. After enrollment and pretesting, participants will be randomly assigned to one of three experimental conditions. Three experimental groups will receive innovative intervention strategies for promoting sun protection practices during vacation – Protection Routine (1), Protection Routine (2) or both Protection Routine (1+2). The fourth experimental condition will be a minimal treatment control group. The primary outcomes of the trial will be a reduction in frequency of sunburn by adherence to the current sun protection advice from the Danish Cancer Society, i.e., use of shade, hats, protective clothing, and sunscreen as secondary outcomes. We hypothesize that the following two interventions will be able to reduce significantly the UVR exposure and risk of sunburns in Danes going on vacation to destinations with high UVI.

**Protection routine 1**

The existing evidence of sun protection against the detrimental effect of UV radiation shows that it is effective if performed correctly **[26, 28]**. We will develop a package of existing training and information and test if it will lead to better adherence. The package will include:

- The rationale of the prioritized sun advice **[32]**
- Activity planner, with suggestions to why a break from the sun is beneficial and how to plan activities before and after UVI maximum i.e. morning/early noon and afternoon/evening **[32]**
- Download of the UV-application ‘UVindeks’ for smartphones developed by the campaign and activating UV alert of geographic destination **[35]**
- A skin type guide

The preferred type of protection of the individual varies so the information and training package in this arm is intended to increase the overall protection level of the individual by advocating several sun protection practices. The sun advice is the most basal sun protection communication **[32]**. Moving outdoor activities away from UV peak hours will theoretical decrease the total UVR received, while maintaining constant outdoor activity level **[32]**. The smartphone application contains information on maximum UVI and cloud cover corrected UVI of any desired location in the world. The cloud cover corrected prognosis makes the information relevant to the recipient as oppose to the standard UVI-max prognosis provided by national weathercasts as these only change slightly through the season **[36]**. Using this intervention will provide participants with relevant information about sun protection at the right time in various geographic locations. In addition, a new alert to implement higher use of the sun advice ‘Shade’ is integrated in the application. The application also includes a skin type test enabling specific messages about outdoor time before individual MED level is reached resulting in sunburn. This means users will receive an alert in the morning, if protection is needed, adapted to the location and UVR exposure to plan their time outdoors and avoid midday exposure. Dissemination of the UV Index alone has not shown to change peoples behavior **[36];** however, it has been able to increase awareness **[37],** which is a precursor to changed behavior in behavioral models **[38]**. Additionally, sun safety smartphone applications have shown indications of improvement in sun protection practices in a randomized trial **[39]**. The skin type guide will also be provided as a paper version for individuals not using the smartphone application. Knowing your skin type is important. Several studies have shown that people overestimate their skin’s thresholds for UVR exposure leading to sunburn **[40]**. This multi-facetted approach adapted to the individual should be able to show a behavioral improvement in sun exposure and protection.

**Protection routine 2**

Two of the largest pitfalls in Danes sun protection is 1) not covering the most skin cancer prone site on the body with a hat and 2) replacing clothing with insufficient amounts of sunscreen.

- A specially designed hat to wear in the sun to test the use of hat when it is available **[33]**
- A newly developed instruction for correct application of sunscreen **[34]**

Denmark is not a hat-wearing society. By making smart design hats available and attractive may increase this sun protection behavior, which has large potential in prevention of skin cancer (**ibid: p2**). A very simple study showed that a brief instruction to sunscreen application increased the provided protection significantly **[34].**

**Protection routine 1+2**

The group will receive both above mentioned interventions and will reveal potential dose response effects from level of protection.

**Control group**

All groups including the control group will receive a minimal intervention with the current sun advice from the Danish Sun Safety Campaign.

**Study population**

The participants of the study are recruited from the Danish civil registration based system. The study population will represent all groups of the Danish population. Eligibility criteria for the project are persons living in Denmark going on vacation in May-December 2018 and having a smartphone compatible with either apple iOS (version 7.0 or newer) or Google Android (version 4.0 or newer). The period May to December is chosen as it includes all types of vacationers. The summer vacation constitutes the majority of the sunny vacations of the Danes. The period includes both the summer, where Danes typically travel to the Mediterranean area and the winter period where travel patterns change a bit to more exotic areas like Thailand, Egypt and the Canarias Islands. Exclusion criteria are persons younger than 18 years of age.

**Randomization**

The participants will be randomized to one of the intervention groups or to the minimal-treatment control group. The randomization procedure is described in the protocol.

**Confounders**

Possible confounders includes age, gender, education, skin type, skin cancer history, nevi, sun-seeking behavior and number of sunny vacations in past 5 years **[28]**.

**Statistical methods and power calculations**

The number of completing participants (n= 1.980) is based on a power calculation, recruitment for similar studies conducted by the applicant **[41, 43]** and our recent pilot study (unpublished, summarized in the end of this application).

A statistical power calculation shows that this sample size yields 95 % probability to detect a 10-percentage point drop from 50 % to 40 % in sunburn between an intervention group and the control group, and 80 % probability to detect a 7-percentage point drop from 50 % to 43 %. Our pilot study showed a 17-percentage point difference between our participants (32 %) relative to our annual sun surveys (49%). The number of invited participants to reach a final sample of ~2000 are included in the flow diagram in the protocol.

To obtain the final study sample size, we assumed 25 % of Danes invited to participate will volunteer for the project **[43]**, >80 % will use of interventions (unpublished pilot study) and > 90 % will complete questionnaire **[41, 43]**. Loss to follow up after one year is expected to be 25 % according to travel patterns with high continuity of the Danes.

Multiple logistic regression analysis will be applied for analysis of differences in sunburn fraction. Analysis of variance will be used to analyze differences in sun protection score and UVR exposure score. Alpha criterion will be p=0.05 (two-tailed).

Intention-to-treat analysis will be applied by using multiple imputation by chained equations to account for missing information on sunburn among non-responders, using PROC MI in SAS 9.3. The imputation procedure includes variables hypothesized to potentially predict missing information (age, sex, education, skin type, sun behaviour, sun protection, sun intentions and intervention group). Analyses will be repeated including responders only and counting non-responders with same sunburn fraction as responders in the control group.

Clustering within families is not included in the planned analysis as it is unlikely to influence the results because participants from the same family have very low odds of being drawn with a total of 30.000 drawn out of the entire Danish population 18+ (app. 4.3 million).

**Evaluation by validated questionnaire**

Sun protective behavior, exposure to UVR and sunburn will be evaluated by use of a questionnaire validated by personal electronic UV-measurements **[7, 44, 45]**. This newly developed survey tool has made it possible in this project to evaluate the skin cancer interventions, without tracking of development of skin cancer which can take several years to develop after excessive UVR exposure. The short time period from intervention to evaluation minimizes recall bias.

**Success criteria**

The primary success criteria of the effectiveness of the interventions is a 10 % decrease in sunburn fraction in the intervention groups as compared to the control group in the post-intervention measurement, as shown possible in the pilot study.

Furthermore, we aim to reach completion and response rates of no less than 90 % of the total sample, which we previously showed to be possible.

Secondary success criteria are increased awareness on the risk of skin cancer, increased use of protection (shade, clothes, hat and sunscreen) and decreased outdoor exposure when the UVI is highest between 12 and 3 pm. For all secondary success criteria, we aim for an improvement of 10 % between interventions and control group.

**Potential bias**

The randomization procedure ensures that all groups are similar; however, there is a risk of bias. The study population could be more or less likely to consist of sun seekers compared to the background population. This will not influence results of the trial because they should be equally distributed across the study arms. However, it could limit generalizability and influence future implementation of the interventions. The validated questionnaire allows us able to adjust for the received UV exposure of the participants in responder analyses.

**Scientific and society perspectives and relevance**

If the developed interventions are effective, they will be used in the Danish Sun Safety Campaign targeting Danes going on vacation to destinations with high UVI and thereby reduce the UVR exposure on a population level and eventually prevent skin cancers in Denmark. In addition, reductions in UVR exposure of the Danes will decrease the economic costs of skin cancer. The costs of skin cancer in Denmark were 250 million DKr annually during 2004-2008 **[46]**. While the return on investment (ROI) has not been estimated for Denmark, in Australia, the ROI for a campaign to prevent skin cancer was estimated to be 230 % **[47].**

**Product and dissemination**

The project aims to produce two scientific papers to document the effect of the study.

In addition, we have planned participation in the 17^th^ World Congress on Cancers of the skin and 5^th^ International Conference on UV and Skin Cancer Prevention. The Danish Sun Safety Campaign has solid experience in disseminating results to the national press and has a person dedicated to the job.

**Expected influence on future research in skin cancer prevention**

The efficacy of sun protection methods is central to skin cancer research. It is estimated that up to 90 % of skin cancers could be prevented by a reduction in UVR exposure **[5]**. However, the evidence of current prevention methods to reduce UVR exposure in practice is insufficient. Thus, it is of utmost importance to establish evidence of current sun protection methods and to develop new methods to improve them where current methods are inadequate. The use of smartphone health applications is widespread and has great potential. However, such health applications, along with personal dosimeters are still poorly documented in the scientific literature. Providing evidence for the use of current and new sun protection methods is leading research worldwide, and there is a large unexploited potential in using smartphones and dosimeters in interventions.

**Organization**

The Danish Sun Safety Campaign is organized in two units, an evaluation unit and a campaign unit. The campaign and the individual members of the campaign has broad experience in setting up interventions **[41, 48, 49]** as well as evaluation **[9, 16-18, 50]** and scientific publication **[10, 11, 29, 41, 43, 46, 48, 49, 51-61]** of results is firmly implemented in the campaign work procedure.

The applicant, Brian Køster, is already a central person in the area and has worked in skin cancer prevention since 2007 to secure and support the scientific evidence **[10, 11, 29, 53-55]** and during 2012-15 as a Ph.D. student to develop improved evaluation to enhance effective campaign efforts **[41, 43, 52, 62]**. The applicant has conducted similar trials during his Ph.D. in the target group and has positive experiences concerning recruitment, consistency, validation and resources to secure high completion.

**National and international network**

In the field of skin cancer prevention behavior research Denmark is a leading country together with Australia. The Australian SunSmart Campaign are important collaborators **[63, 64]**. Australian sun behavior is culturally very different from the behavior among northern Europeans. Key players in Denmark, and worldwide are the UVR research

group headed by Dr. Wulff at Bispebjerg Hospital and the Danish Sun Safety Campaign. This project is developing further the clinical research from Dr. Wulff’ group **[65-68]**, the population-based research on sun protection in warm-weather resorts **[69, 70]** and the use of smartphone technology for promoting sun protection **[39, 71, 72]** by Dr. Buller’s group and the population based intervention efforts of the Danish Sun Safety campaign **[8, 10, 29, 73]**.

**Summary of completed pilot studies securing base assumptions**

We have previously conducted feasibility and intervention studies demonstrating the feasibility of this study. We have shown a recruitment rate of 25 % when sampled from the Danish civil registration system **[43]**. We have also shown that the completion rate in this sample among the recruited participants were above 90 % **[41, 43]**.

We have now piloted the preliminary planned interventions in a small study of 147 persons aged 15-65 representing the Danish population in gender, age, education and geography, going on vacation to a country with a higher uv-index (UVI 8+) than Denmark in the summer of 2016 (not published). They were recruited from a web panel during July 2016 and were vacationing for 1-2 weeks in July-mid August. The participants were introduced to the majority of the elements in intervention 1. The feasibility of intervention 2 was previously shown **[41, 62].** Ninety-three percent received and read the material. At posttest, we showed a sunburn rate in the study population, which was 17 percentage points lower than reported for our annual survey of the Danish population. All intervention elements (Sun advice, smartphone application, sunscreen application guide, skin type guide) proposed in this study were considered relevant and useful by more than 80 % of those who completed the pilot study. The skin type guide scored lowest with 81 % and the app scored highest with 95 % among those who had it available (google android and apple ios phones; not available for windows phones for instance). Furthermore, we saw a 9-percentage point increase in use of sunscreen, while use of long-sleeves and hat was largely unchanged. The hats proposed for the study, were not tested in the pilot, however the feasibility of the hat was previously tested in minor interventions. We also show a slight increase in knowledge of the UV Index in the population survey from 89 % to 93 % in the pilot test.

**Potential pitfalls**

Albeit highly unlikely, we have sketched various scenarios that could influence the project.

The project is based in the Danish Cancer Society, which means that during the project period, should any of the applicants change job, be long term absent/sick or likewise, qualified researchers of same level will replace them.

The project involves products from third part companies, which can be prone to internal or external structural changes or bankruptcy, leaving them no possibility to deliver a given product. In any case, an equally qualified product will be sought in replacement.

The recruitment method is register based. Should the Danish Civil Registry for some reason not be able to deliver data, like sudden legal changes in person data handling or likewise, other methods of recruitment are available.

All potential changes to the project will of course be reported to funder immediately.

**Protocol**

**Time Schedule**

|  |  |  |
| --- | --- | --- |
| Mar-Apr 2018 | Planning of intervention & evaluation | Registering trial  Ordering sample from civil registry system  Preparation of interventions  Preparation of information and homepage |
| Apr-May 2018 | Recruitment and scheduling; | Sending invitations  Ordering products for intervention  Baseline Questionnaire |
| May-Dec 2018 | Execution of interventions | Adjusting intervention groups according to participation  Sending intervention packages for participants  Sending questionnaires for post evaluation |
| Jun 2017 -Feb 2019  Jun - Nov 2019 (follow-up) | Data collection and data handling | Evaluation of participation and possible reminders  Conversion of questionnaire data files for SAS and Purification of data  Recording of relevant UV Indexes from vacation destinations |
| May-Dec 2019 | 1-year Follow-up | Recruitment and scheduling  Sending questionnaires for evaluation |
| May-Aug 2019 | Evaluation and documentation | Analysis and manuscript 1 (baseline) |
| Aug-Nov 2019 |  | Analysis and manuscript 2 (main study) |
| Dec 2019 - Mar 2020 |  | Analysis and manuscript 3 (follow-up) |
| Aug 2019 - Sep 2020 | Dissemination of results and implementation of successful interventions | Publications in national press and international scientific papers  Update of guidelines |

**Registering trial**

The trial will be registered at clinicaltrials.gov.

**Civil registry sample**

The sample description of the trial to Danish health data board will be developed. Permissions will be gathered from The Danish Data Protection Agency and the National Committee of Health Research Ethics. A sample of 25.400 individuals aged 18 and older will be requested.

**Study population recruitment**

The study population will be recruited from the Danish Civil Registry System. The aim is to obtain a sample representing all groups of the Danish population. Recruitment for this type of trial is difficult as the target population is not easy to reach without selection bias. To improve recruitment rates invitations will be targeted to the person in the sample or to any family member in the household aged 18 and above. We aim to receive full data for a final population of approximately 2.000 persons.

It may appear, as 8 % of the original draw in the final data is not much; however, as less than half of the Danes are having vacation abroad every year it corresponds to a reach of 18 % among those eligible for the study. Alternative recruitment approaches like airport recruiting most likely would not decrease any bias or yield a higher percentage of recruited travelers. In addition, Danes also departure from nearby airports in Germany and Sweden, which would be difficult to include in the project.

**Planned evaluation**

The evaluation will be conducted in the online survey system, SurveyXact, provided by Rambøll. The system is used in numerous evaluations of the Danish Cancer Society. The evaluation is based on the validated UV behavior questionnaire recently developed by Danish Sun Safety Campaign. The questionnaire will be adapted to the trial.

**Preparation of interventions**

An intensive intervention package was developed prior to the study. The package is composed by known elements from the Danish Sun Safety campaign. The package also includes download of the UV-App developed by the Danish Sun Safety Campaign. The app includes an alert function for high UV-Indexes and can be adapted to the correct location by GPS or a selected city around the world. This mobile app is fully developed and available for this trial. The participants randomized to the package will receive alerts to take precautions against solar UVR before and during their vacation.

**Invitations**

The invitations for the project will be handled (printed, enveloped and sent) by trained volunteers from the Danish Cancer Society. Participants will sign up for the project on a developed web-page of the project. On the project homepage the participants will fill in a short form of contact information, demographic information, departure and arrival dates of their upcoming vacation and baseline sun behavior information for non-responder analyses. Participants who have not yet planned their vacation are instructed to contact the project team

**Flow Diagram**

## Enrollment

Invitations to random sample of Danes aged 15 years and above from the Civil Registration System, n=25.400

Persons not eligible, not going abroad, n=14.200

Invitations to persons eligible, going on vacation, n=11.200

Agree to participate, n=2.800, 25 %

Complete baseline questionnaire

Unavailable in study period/ withdrawal of participation n= 76

Intervention groups

n=2.043, 3*681

## Available participants randomized to intervention or control, n=2.724

Control group, n= 681

## Randomized Provisional sample, n=2.500

Protection routine 1+2

n=625

Protection routine 2

n=625

Protection routine 1

n=625

Control

(n=625)

## Provisional completed sample, n=2.000

**Completed post-test**

Drop out from intervention and control groups, n = 224

Completion rate including questionnaire among participants aims to be >= 80 % (based on rates from similar Ph.D. project (89-93 %) )

Total non-responders from completed intervention and control groups, n = 500

Total randomized non-responders, n = 724

once they have planned it. Likewise they will be reminded every 2-3 weeks during the period by mail or phone. Repeated invitations will be sent after two and four weeks to non-responders. Afterwards, participants will be contacted by the volunteers by phone to confirm their participation and receive information about the study. A student will be dedicated in organizing workflow of the volunteers and participate in the training of the volunteers. We have good experiences with this procedure from other projects like [www.mituv.dk](http://www.mituv.dk) and [www.statusd.dk](http://www.statusd.dk) also funded by TrygFonden.

Invitations will be identical for all invited potential participants. It will include invitation to participate in a project about how the Danish Cancer Society can improve their knowledge about sun protection information and that they will be allocated to use a specific sun protection during their vacation. It will also include information about which procedures may be applied.

**Recruitment**

The invited participants will provide their contact data and supplemental background data upon recruitment on the project homepage. Only one person from a family will be enrolled in the study.

**Development of protection routines and training package intervention**

The final information package is composed by various elements from the Danish Sun Safety campaign as described in the project description. The package was created by an experienced group from the Danish Sun Safety Campaign, including experts in public health, anthropology and communication and was be piloted before the trial.

**Main study**

The participants will be randomized to one of the three groups below after agreeing to participate. The detailed distribution of participants are shown in the flow diagram. The randomization will be stratified by gender (Male/Female), age group (18-25, 26-35, 36-45, 46-55, 56-65 and 65+) and region (Capital, Zealand, Northern Jutland, Central Jutland and Southern Denmark). The randomization procedure will be blind to the researcher. All participants will be contacted immediately before their vacation and will receive their intervention package before departure.

**Control group – Minimal intervention**

All groups including the control group will receive a minimal intervention with the sun advice from the Danish Sun Safety Campaign.

**Protection Routine 1 – Intensive information and training in current sun advice**

The intensive information package is described in the project description. The newly developed information folder, includes the sun advice of the Danish Sun Safety Campaign, the rationale behind the prioritization and an overview of preventive measures against the sun. The activity planner includes examples of outdoor activities like bathing, sports and excursions before and after noon (UVI maximum) and indoor activities like shopping, lunch, relaxation and transportation in the middle of the day and possibility to plan days in the vacation to minimize risk. The instructions for download of the UV-App includes use of the alert, and information about the features of the app; e.g. how to be informed of current UV risk before and during vacation. In addition, link for download of the app will be sent by both text-message and e-mail. The skin type guide is included in the app and comes as an individual entity. It includes scoring questions about tan and burn reaction in the sun, a color scale, eye and hair color and freckles. Afterwards the participants get a description of their skin type and examples of how short time their particular skin type will resist the sun before erythema occurs at particular UV-levels and functions as an eye-opener. The participants will receive a physical copy of the material by mail and an electronic version by e-mail to be able to revisit it.

**Protection Routine 2 – Thorough sunscreen and availability of hat**

The sunscreen application instruction is based on a recently developed thorough application instructions for use of sunscreen to counter the described problems and deficits of current sunscreen use. It describes every part of the body, the needed volume for that part and application patterns. The package will also include a hat specifically designed for protection and comfort and the availability of a hat will function to increase the use of hat. The hat is simple bucket hat version with a brim, cheap, in light fabric and foldable for easy packing.

**Protection Routine 1 & 2 – Combined**

As described above

**Completion of intervention**

While it is hoped that all individuals will use the interventions, research by our team and others have found that exposure to interventions typically vary across participants. Exposure to the interventions will be measured by post-test questionnaire. Also, use of the mobile app will be assessed by registering when downloaded from a link sent by email and text-message reminders to the participants. Automated email and text-message reminders will be considered as tools to increase use of the mobile app and the personal dosimeter. Completion of the full intervention will be defined as follows: control group - having received the minimal intervention materials; Protection Routine 1- use of 50 % or more of the intervention materials in the package; Protection Routine 2 – . However, we will also analyze the amount of use of the interventions for the impact of intervention exposure on intervention effectiveness.

**Data collection and handling**

Data collection is executed by an electronic questionnaire sent immediately after return from the vacation. If unanswered reminders are sent after 4 days, 9 days and 14 days. The data collection methods follows results of a recent validation study, which showed that validity of the questionnaire answers is crucially dependent on short time from exposure to answering the questionnaire. Responses within 2 weeks of return from vacation were valid.

**1-year-follow-up**

Participants that have completed the intervention are invited to a follow up study the following year. The procedure is similar to the initial procedure. Only now, participants are invited by e-mail (supported by phone) and screened for possible vacation to a sunny destination. Participants going on a sunny vacation will receive a questionnaire immediately after return from the vacation for evaluation of long-term effects.

1. Bray, F., et al., *Global estimates of cancer prevalence for 27 sites in the adult population in 2008.* Int.J.Cancer, 2012.

2. Engholm, G., et al., *NORDCAN: Cancer Incidence, Mortality, Prevalence and Prediction in the Nordic Countries, Version 5.1. Association of Nordic Cancer Registries.Danish Cancer Society.* 2012.

3. Armstrong, B.K. and A. Kricker, *How much melanoma is caused by sun exposure?* Melanoma Res., 1993. **3**: p. 395-401.

4. Gandini, S., et al., *Meta-analysis of risk factors for cutaneous melanoma: II. Sun exposure.* Eur.J.Cancer, 2005. **41**(1): p. 45-60.

5. Lucas, R.M., et al., *Estimating the global disease burden due to ultraviolet radiation exposure.* Int.J.Epidemiol., 2008. **37**(3): p. 654.

6. Doll, R. and R. Peto, *The causes of cancer: quantitative estimates of avoidable risks of cancer in the United States today.* J.Natl.Cancer Inst., 1981. **66**(6): p. 1191-1308.

7. Thieden, E., *Sun exposure behaviour among subgroups of the Danish population. Based on personal electronic UVR dosimetry and corresponding exposure diaries.* Dan.Med.Bull., 2008. **55**(1): p. 47-68.

8. Krarup, A., F, C. Thorgaard, and C.L. Behrens, *Danskernes solvaner på solferier 2010 (Report in Danish accessed at* [*www.skrunedforsolen.dk*](http://www.skrunedforsolen.dk)*)*. 2010. p. 1-17.

9. Behrens, C.L.D., L., *Danskernes solvaner i den danske sommer 2013(Report in Danish accessed at* [*www.skrunedforsolen.dk*](http://www.skrunedforsolen.dk)*)*

2014.

10. Koster, B., et al., *Prevalence of sunburn and sun-related behaviour in the Danish population: A cross-sectional study.* Scand. J. Public Health, 2010. **38**(5): p. 548-552.

11. Koster, B., et al., *Sunbed use and campaign initiatives in the Danish population, 2007-2009: a cross-sectional study.* J. Eur. Acad. Dermatol. Venereol., 2011. **25**(11): p. 1351-1355.

12. Parisi, A.V., et al., *Personal exposure distribution of solar erythemal ultraviolet radiation in tree shade over summer.* Phys.Med.Biol., 2000. **45**(2): p. 349-356.

13. Aguilera, J., et al., *New advances in protection against solar ultraviolet radiation in textiles for summer clothing.* Photochem Photobiol, 2014. **90**(5): p. 1199-206.

14. Kimlin, M.P., A V. *Ultraviolet protective capabilities of hats under two different atmospheric conditions*. 1999 23. Aug. 2016].

15. Petersen, B. and H.C. Wulf, *Application of sunscreen--theory and reality.* Photodermatol.Photoimmunol.Photomed., 2014. **30**(2-3): p. 96-101.

16. Behrens, C.L.M., M.K.H.; Jensen, M.P.; Christensen, A.S., *Danskernes solvaner i den danske sommer 2014. Kræftens Bekæmpelse og TrygFonden smba (TryghedsGruppen smba) 2015*. 2015: [www.skrunedforsolen.dk](http://www.skrunedforsolen.dk).

17. Behrens, C.L.S., C., *Danskernes solarievaner 2014 – en kortlægning. Kræftens Bekæmpelse og TrygFonden smba (TryghedsGruppen smba) 2015*. 2015: [www.skrunedforsolen.dk](http://www.skrunedforsolen.dk).

18. Meyer, M.K.H.B., C.L., *Danskernes solvaner på solferie 2014 – en kortlægning. Kræftens Bekæmpelse og TrygFonden smba (TryghedsGruppen smba) 2015*. 2015: [www.skrunedforsolen.dk](http://www.skrunedforsolen.dk).

19. Petersen, B., et al., *A sun holiday is a sunburn holiday.* Photodermatol.Photoimmunol.Photomed., 2013. **29**(4): p. 221-224.

20. Petersen, B., et al., *Determinants of personal ultraviolet-radiation exposure doses on a sun holiday.* Br.J.Dermatol., 2013. **168**(5): p. 1073-1079.

21. Petersen, B., et al., *Validation of self-reported erythema: comparison of self-reports, researcher assessment and objective measurements in sun worshippers and skiers.* J.Eur.Acad.Dermatol.Venereol., 2012.

22. Pettijohn, K.J., et al., *Vacations to waterside locations result in nevus development in Colorado children.* Cancer Epidemiol.Biomarkers Prev., 2009. **18**(2): p. 454-463.

23. O'Riordan, D.L., et al., *A day at the beach while on tropical vacation: sun protection practices in a high-risk setting for UV radiation exposure.* Arch.Dermatol., 2008. **144**(11): p. 1449-1455.

24. Sciøth, C.K., B., *The Danes sun habits during their summer vacation in 2015 (in Danish)*. 2016.

25. Osterlind, A., K. Hou-Jensen, and O. Moller Jensen, *Incidence of cutaneous malignant melanoma in Denmark 1978-1982. Anatomic site distribution, histologic types, and comparison with non-melanoma skin cancer.* Br J Cancer, 1988. **58**(3): p. 385-91.

26. Green, A.C., et al., *Reduced Melanoma After Regular Sunscreen Use: Randomized Trial Follow-Up.* J.Clin.Oncol., 2011. **29**(3): p. 257-263.

27. Green, A., et al., *Daily sunscreen application and betacarotene supplementation in prevention of basal-cell and squamous-cell carcinomas of the skin: a randomised controlled trial.* Lancet, 1999. **354**(9180): p. 723-729.

28. IARC, *Monograph 100 D - Solar and ultraviolet radiation*. 2011: Lyon, France. p. 35-102.

29. Køster, B., *Issues to address when investigating sunscreen use.* British Journal of Dermatology, 2015. **172**(2): p. 321-2.

30. Autier, P., *Sunscreen abuse for intentional sun exposure.* Br.J.Dermatol., 2009. **161 Suppl 3**: p. 40-45.

31. Petersen, B., et al., *Sunscreen use and failures--on site observations on a sun-holiday.* Photochem.Photobiol.Sci., 2013. **12**(1): p. 190-196.

32. Greinert, R., et al., *European Code against Cancer 4th Edition: Ultraviolet radiation and cancer.* Cancer Epidemiol, 2015. **39 Suppl 1**: p. S75-83.

33. Østerlind, *The danish case-control study of cutaneou malignant melanoma II. Importance of UV-light exposure.* Int.J.Cancer, 1988: p. 319.

34. Jeanmougin, M., A. Bouloc, and J.L. Schmutz, *A new sunscreen application technique to protect more efficiently from ultraviolet radiation.* Photodermatol Photoimmunol Photomed, 2014. **30**(6): p. 323-31.

35. Society, D.C. *UV Indexs application*. 25. Aug. 2016].

36. Allinson, S., et al., *Validity and use of the UV index: report from the UVI working group, Schloss Hohenkammer, Germany, 5-7 December 2011.* Health Phys., 2012. **103**(3): p. 301-306.

37. Italia, N. and E.A. Rehfuess, *Is the Global Solar UV Index an effective instrument for promoting sun protection? A systematic review.* Health Educ.Res., 2012. **27**(2): p. 200-213.

38. Bartholomew, L.K., et al., *Planning Health Promotion Programs: An Intervention Mapping Approach*. 2011.

39. Buller, D.B., et al., *Evaluation of immediate and 12-week effects of a smartphone sun-safety mobile application: a randomized clinical trial.* JAMA Dermatol, 2015. **151**(5): p. 505-12.

40. Reeder, A.I., V.A. Hammond, and A.R. Gray, *Questionnaire items to assess skin color and erythemal sensitivity: reliability, validity, and "the dark shift".* Cancer Epidemiol.Biomarkers Prev., 2010. **19**(5): p. 1167-1173.

41. Koster, B., et al., *Effects of smartphone diaries and personal dosimeters on behavior in a randomized study of methods to document sunlight exposure.* Prev Med Rep, 2016. **3**: p. 367-72.

42. Dobbinson, S.J.W., M.A.; Jamsen, K.M.; Herd, N.L.; Spittal, M.J.; Lipscomb, J.E.; Hill, D.J., *Weekend sun protection and sunburn in Australia -Trends (1987-2002) and association with sunsmart television advertising.* Am.J.Prev.Med., 2008. **34**(2): p. 94-101.

43. Koster, B., et al., *The validated sun exposure questionnaire - Association of objective and subjective measures of sun exposure in a Danish population based sample.* Br J Dermatol, 2016.

44. Koster, B., et al., *Feasibility of smartphone diaries and personal dosimeters to quantitatively study exposure to ultraviolet radiation in a national sample.* Photodermatology, photoimmunology & photomedicine, 2015. **31**: p. 252-260.

45. Allen, M. and R. McKenzie, *Enhanced UV exposure on a ski-field compared with exposures at sea level.* Photochem.Photobiol.Sci., 2005. **4**(5): p. 429-437.

46. Bentzen, J., et al., *Costs of illness for melanoma and nonmelanoma skin cancer in Denmark.* Eur.J.Cancer Prev., 2013.

47. Gordon, L.G. and D. Rowell, *Health system costs of skin cancer and cost-effectiveness of skin cancer prevention and screening: a systematic review.* Eur.J.Cancer Prev., 2014.

48. Dalum, P., et al., *A cluster randomised controlled trial of an adolescent smoking cessation intervention: Short and long-term effects.* Scand.J.Public Health, 2012. **40**(2): p. 167-176.

49. Bentzen, J., et al., *Determinants of sunbed use in a population of Danish adolescents.* Eur.J.Cancer Prev., 2013. **22**(2): p. 126-130.

50. Darsø, L., C.L. Behrens, and L.H. Schmidt, *Børn og unges solarievaner 2010 - en kortlægning*. 2011. p. 1-61.

51. Hansen, M.R. and J. Bentzen, *High-risk sun-tanning behaviour: a quantitative study in Denmark, 2008-2011.* Public Health, 2014. **128**(9): p. 777-83.

52. Koster, B. and J.N. Søndergaard, J.B. ;;Christensen, K.B. ;; Allen, M. ;; Olsen, A. ;; Bentzen, J., *The validated sun exposure questionnaire - Knowledge deficit, attitude and behavior scales for skin cancer prevention and association to objectively measures of sun exposure and sunburn in a Danish population based sample.* In Review, 2016.

53. Koster, B., et al., *Vacations to sunny destinations, sunburn, and intention to tan: a cross-sectional study in Denmark, 2007-2009.* Scand.J.Public Health, 2011. **39**(1): p. 64-69.

54. Krarup, A.F., et al., *Sunbed use by children aged 8-18 years in Denmark in 2008: a cross-sectional study.* Br.J.Dermatol., 2011. **165**(1): p. 214-216.

55. Koster, B., et al., *Sunbed use in the Danish population in 2007: a cross-sectional study.* Prev Med, 2009. **48**(3): p. 288-90.

56. Behrens, C.L., et al., *Sunburn in children and adolescents: associations with parents' behaviour and attitudes.* Scand.J.Public Health, 2013. **41**(3): p. 302-310.

57. Dalum, P., et al., *The Systematic Development of an Internet-Based Smoking Cessation Intervention for Adults.* Health Promot Pract, 2016. **17**(4): p. 490-500.

58. Skov-Ettrup, L.S., et al., *Reach and uptake of Internet- and phone-based smoking cessation interventions: results from a randomized controlled trial.* Prev Med, 2014. **62**: p. 38-43.

59. Skov-Ettrup, L.S., et al., *The effectiveness of telephone counselling and internet- and text-message-based support for smoking cessation: results from a randomized controlled trial.* Addiction, 2016. **111**(7): p. 1257-66.

60. Andersen, A., et al., *Design of a school-based randomized trial to reduce smoking among 13 to 15-year olds, the X:IT study.* BMC Public Health, 2014. **14**: p. 518.

61. Skov-Ettrup, L.S., et al., *Comparing tailored and untailored text messages for smoking cessation: a randomized controlled trial among adolescent and young adult smokers.* Health Educ Res, 2014. **29**(2): p. 195-205.

62. Koster, B., et al., *Feasibility of smartphone diaries and personal dosimeters to quantitatively study exposure to ultraviolet radiation in a small national sample.* Photodermatol Photoimmunol Photomed, 2015. **31**(5): p. 252-60.

63. Chodick, G., et al., *Agreement between diary records of time spent outdoors and personal ultraviolet radiation dose measurements.* Photochem.Photobiol., 2008. **84**(3): p. 713-718.

64. Sun, J., et al., *The relationship between ambient ultraviolet radiation (UVR) and objectively measured personal UVR exposure dose is modified by season and latitude.* Photochem Photobiol Sci, 2014. **13**(12): p. 1711-8.

65. Bech-Thomsen, N. and H.C. Wulf, *Sunbathers' application of sunscreen is probably inadequate to obtain the sun protection factor assigned to the preparation.* Photodermatol.Photoimmunol.Photomed., 1992. **9**(6): p. 242-244.

66. Faurschou, A. and H.C. Wulf, *Durability of the sun protection factor provided by dihydroxyacetone.* Photodermatol.Photoimmunol.Photomed., 2004. **20**(5): p. 239-242.

67. Petersen, A.B., R. Na, and H.C. Wulf, *Sunless skin tanning with dihydroxyacetone delays broad-spectrum ultraviolet photocarcinogenesis in hairless mice.* Mutat Res, 2003. **542**(1-2): p. 129-38.

68. Petersen, A.B., et al., *Dihydroxyacetone, the active browning ingredient in sunless tanning lotions, induces DNA damage, cell-cycle block and apoptosis in cultured HaCaT keratinocytes.* Mutat Res, 2004. **560**(2): p. 173-86.

69. Andersen, P.A., et al., *Environmental variables associated with vacationers' sun protection at warm weather resorts in North America.* Environ Res, 2016. **146**: p. 200-6.

70. Buller, D.B., et al., *Rationale, design, samples, and baseline sun protection in a randomized trial on a skin cancer prevention intervention in resort environments.* Contemp Clin Trials, 2016. **46**: p. 67-76.

71. Buller, D.B., et al., *Smartphone mobile application delivering personalized, real-time sun protection advice: a randomized clinical trial.* JAMA Dermatol, 2015. **151**(5): p. 497-504.

72. Buller, D.B., et al., *User-centered development of a smart phone mobile application delivering personalized real-time advice on sun protection.* Transl Behav Med, 2013. **3**(3): p. 326-34.

73. Krarup, A.F., et al., *Sun exposure habits of the Danes in the Danish summer 2010 (Report in Danish accessed at* [*www.skrunedforsolen.dk*](http://www.skrunedforsolen.dk)*). Solundersøgelsen 2010 - En kortlægning af danskernes solvaner i den danske sommer*. 2011. p. 1-59.
