# Supplementary material for: Novel sunprotection interventions to prevent skin cancer: A randomized study targeting Danes going on vacation to destinations with high UV index
Source: PLoS One. 2020 Dec 31;15(12):e0244597. doi: 10.1371/journal.pone.0244597 (PMC7774952; doi:10.1371/journal.pone.0244597)
Supplement: S2 File — (PDF) [file pone.0244597.s004.pdf]

18. JANUAR 2017

Hvad skal jeg anmelde?

Sundhedsvidenskabelige forskningsprojekter skal anmeldes til en videnskabsetisk komité. Nedenfor kan du se hvilke forskningsprojekter, der skal og ikke skal anmeldes.

Sundhedsvidenskabelige forskningsprojekter skal anmeldes til en videnskabsetisk komité. Komiteen foretager i tvivlstilfælde en konkret vurdering af anmeldelsespligten i henhold til lov om videnskabsetisk behandling af sundhedsvidenskabelige forskningsprojekter (komitéloven).

Anmeldelsespligten omfatter:

- Forsøg på levendefødte menneskelige individer, menneskelige kønsceller, der agtes anvendt til befrugtning, menneskelige befrugtede æg, fosteranlæg og fostre, væv, celler og arvebestanddele fra mennesker, fostre og lign. eller afdøde (komitélovens § 2, nr. 1).

- Kliniske forsøg med lægemidler på mennesker, dvs. forsøg på mennesker, der har til formål at afdække de kliniske, farmakologiske eller andre farmakodynamiske virkninger af et eller flere forsøgslægemidler eller identificere bivirkninger ved et eller flere forsøgslægemidler eller at undersøge absorption, distribution, metabolisme eller udskillelse af et eller flere forsøgslægemidler med henblik på at vurdere sikkerhed eller effekt af lægemidlet (komitélovens § 2, nr. 2).

- Klinisk afprøvning af CE-mærket og ikke CE-mærket medicinsk udstyr, dvs. forsøg på mennesker, der har til formål at afdække eller efterprøve sikkerheden eller ydeevnen af medicinsk udstyr (komitélovens § 2, nr. 3). Se også Lægemiddelstyrelsens side om klinisk afprøvning af medicinsk udstyr.

  Medicinsk udstyr er udstyr til at undersøge, overvåge, behandle eller lindre sygdom hos mennesker, fx sprøjter, operationsudstyr, hospitalssenge, pacemakere, hofteimplantater, krykker eller kondomer.

Nedenstående skal ikke anmeldes:

- Spørgeskemaundersøgelser og interviewundersøgelser, som ikke omfatter menneskeligt biologisk materiale (komitélovens § 14, stk. 2).
- Registerforskningsprojekter, som ikke omfatter menneskeligt biologisk materiale, men er baseret på rene data, dvs. bogstaver, tal, tegn osv. (komitélovens § 14, stk. 2). Se her om anmeldelse til andre myndigheder.

- Forskningsprojekter, hvor der alene indgår anonymt menneskeligt biologisk materiale, der er indsamlet i overensstemmelse med lovgivningen på indsamlingsstedet, dvs. materiale, der irreversibelt hverken direkte eller indirekte kan henføres til bestemte personer (undtagelse gælder dog forskningsprojekter nævnt i § 25 i lov om assisteret

reproduktion i forbindelse med behandling, diagnostik og forskning mv., som skal anmeldes).

- Forsøg på cellelinjer el.lign., der stammer fra et godkendt forsøg med indsamling af celler eller væv, og som har opnået den nødvendige godkendelse (undtagelse gælder dog, hvis forsøget angår anvendelse af befrugtede æg, stamceller og stamcellelinjer herfra, som nævnt i § 25 og § 27, stk. 2 i lov om assisteret reproduktion i forbindelse med behandling, diagnostik og forskning mv., som skal anmeldes).

- Ikke-interventionsundersøgelser med lægemidler, der er defineret i bekendtgørelse om god klinisk praksis i forbindelse med kliniske forsøg med lægemidler på mennesker § 2, nr. 5.

- Patientbehandling eller behandlingsforsøg, se nedenfor.

- Kvalitetssikring eller kvalitetskontrol, se nedenfor.

Hvad er sundhedsvidenskabelig forskning?

Sundhedsvidenskab omfatter såvel behandling, undersøgelse **og** forebyggelse som rehabilitering og vil derfor kunne indfange, at der inden for alle typer af sundhedsvidenskabelig forskning udføres forsøg, som kan indeholde relevante videnskabsetiske aspekter.

Sundhedsvidenskabelig forskning omhandler primært lægevidenskab, farmaceutisk videnskab, sygeplejevidenskab, tandlægevidenskab mv. Der anvendes fx biologiske, kliniske, epidemiologiske, socialmedicinske og psykologiske forskningsmetoder.

Udvalget om Revision af det Videnskabsetiske Komitésystem, Sundhedsministeriet, 2010 foreslår i Betænkning nr. 1515, at ”anmeldelsespligtig forskning afgrænses som sundhedsvidenskabelig interventionsforskning. Herved synliggøres det, at der i de anmeldelsespligtige forsøg skal indgå en forsøgsperson (eller materiale fra en sådan), der udsættes for en intervention, som i langt de fleste tilfælde vil dreje sig om behandling, undersøgelse eller forebyggende indgreb”.

Sundhedsvidenskabelig forskning har således til formål at skabe ny viden eller efterprøve eksisterende viden om fx:

- sygdoms opståen eller behandling, diagnostik, forebyggelse, rehabilitering af mennesker samt
- menneskets biologiske, fysiologiske eller psykologiske processer og arveanlæg.

For at der er tale om en et anmeldelsespligtigt sundhedsvidenskabeligt forskningsprojekt, skal projektet altså både have et sundhedsvidenskabeligt formål og intervention.

Patientbehandling

Forebyggelse, diagnostik og behandling mv. over for en konkret person skal ikke anmeldes. Behandlingsforsøg, hvor lægen med skærpet informeret samtykke tager ansvar for en forsøgsbehandling af en konkret, kritisk syg patient som en ”sidste udvej”, er heller ikke anmeldelsespligtige.

Af Sundhedsstyrelsens Vejledning nr. 11052 af 2. juli 199 om indførelse af nye behandlinger i sundhedsvæsenet fremgår: ”Hvor der foreligger et – implicit eller eksplicit – hypoteseafprøvende og vidensgenererende element i relation til valg af behandlingsmetode, bliver patienten i relation til behandlingen ikke blot et "mål", men også et "middel", nemlig et middel til opnåelse af ny viden. Der er således ikke – specielt ikke set med patientens øjne – et klart og entydigt sammenfald mellem patientens konkrete interesse i et godt behandlingsresultat og lægens interesse i behandlingens anvendelse. Det er dette, som nødvendiggør en videnskabsetisk vurdering af fremgangsmåden, og dermed at fremgangsmåden formaliseres og anmeldes”.

Hvis der er tvivl om, hvorvidt nye behandlingsmetoder skal anmeldes som et forskningsprojekt eller ej, er det afgørende at se på formålet. Hvis formålet er at generere ny viden ud over at behandle patienten, så skal projektet anmeldes.

Det er ikke nødvendigt at anmelde et projekt til en videnskabsetisk komité eller udarbejde en protokol, hvis der findes omfattende kliniske erfaringer, der på en overbevisende måde belyser effekt, bivirkninger og risici ved behandlingen. Den ansvarlige læge skal derfor overveje, om der foreligger tilstrækkelig evidens for at indføre behandlingen. Hvis der er tvivl, anmeldes forsøget til det videnskabsetiske komitésystem. Dette gælder også, hvis der er tvivl om de faglige resultater, der ligger til grund for at indføre en ny behandling.

Kvalitetskontrol og kvalitetsudvikling

Kvalitetskontrol og kvalitetsudvikling skal ikke anmeldes til videnskabsetisk komité.

Vejledning nr. 11052 af 2. juli 199 om indførelse af nye behandlinger i sundhedsvæsenet anfører om kvalitetskontrol: ”En kvalitetskontrol sigter ikke på at opnå ny viden om behandlingens værdi, men afprøver den kliniske enheds funktion”.

Kvalitetsudvikling er udvikling af nye metoder eller nye indikatorer inden for allerede etablerede områder. Det giver ny viden lokalt i organisationen, men den er ikke generaliserbar uden for denne.

Kvalitetskontrol eller kvalitetsudvikling er typisk aktiviteter, der indgår i sundhedsvæsenets drift vedrørende fx en sygehusafdelings opnåede behandlingsresultater for en given patientgruppe. Der kan være tale om sammenligning af en aktuel behandling med den vedtagne behandlingsinstruks, evaluering af effekt og omkostninger af forskellige behandlingsprincipper. Der vil ofte være tale om retrospektive eller observerende prospektive undersøgelser, hvor der ikke interveneres på behandlingen mv.

Det samme gælder en virksomheds produktudvikling, fx kalibrering af et apparat med blod. Formålet er her ikke at opnå ny viden, men at afprøve en sygehusafdelings kliniske funktion eller et produkts effekt.

Forskning i alternative terapiformer

Forskning i alternativ behandling kan være anmeldelsespligtig, såfremt de ovenfor nævnte formål er opfyldt. Fx har forsøg med mindfulness været kendt anmeldelsespligtige i komitésystemet. Selv om selve interventionen kan karakteriseres som alternativ, er kravene til veldefinerede inklusions- og

eksklusionskriterier og effektmål usvækkede, og interventionerne skal være reproducerbare.

Offentlig eller privat regi

Projekterne kan udføres i offentligt regi, herunder i en region, kommune eller universitet eller i privat regi. Projekter om forebyggelse i kommunerne, hvor der sker evaluering, kan være sundhedsvidenskabelig forskning — fx hvis man ønsker at få mere at vide om hvilke indsatser, der er effektive i forhold til årsager til sygdom, og den sundhedsfremmende indsats udvides med videnskabelig metode/måling for at opnå generaliserbar viden om sundhedsmæssig effekt. Se mere herom.

Forskningsaktivitet i Danmark

Det er kun forskningsaktivitet i Danmark, udført af en forsøgsansvarlig med arbejdssted i Danmark, som skal anmeldes til en videnskabsetisk komité.

Et projekt kan overskride grænser, enten ved at forsøgspersoner rejser ud eller ind i landet, eller ved at biologisk materiale importeres/eksporteres. Hvis forsøgspersoner fx bliver sendt til udlandet til videre undersøgelse eller udredning i et forsøg, vil der sideløbende skulle ske anmeldelse til den relevante videnskabsetiske komité i udlandet, da den danske komité ikke har kompetence til at fastsætte vilkår for aktiviteter i udlandet. Den danske komité kan dog undlade at godkende forskningsaktiviteten i Danmark, hvis der ikke er oplyst om en tilfredsstillende beskyttelse af forsøgspersonernes sikkerhed, rettigheder og velfærd i tilknytning til det videre forløb i udlandet. Tilsvarende gælder ved indrejse i et udenlandsk forsøg. Et forskningsprojekt med fx importeret personhenførbart biologisk materiale skal også anmeldes. Afgørende er, at forskningsaktiviteten finder sted i Danmark.

Ønsker en forsøgsansvarlig at forske i Danmark i biologisk materiale fra udlandet (fx fra en klinisk biobank i udlandet) med det formål at skabe ny viden om fx et sygdomsområde mv., skal det anmeldes til den regionale videnskabsetiske komité i Danmark.

Er der imidlertid tale om, at en dansk forsker eller en laboratorieafdeling udfører analyse af biologisk materiale fra udlandet på vegne af en forsker eller et firma, der har sit virke i udlandet, skal dette **ikke** anmeldes. I en sådan situation foregår forskningen ikke i Danmark, når der blot er tale om en laboratorieydelse el.lign., hvor resultaterne returneres til forskeren eller firmaet i udlandet.

Den kompetente videnskabsetiske komité

For uddybende information om, hvilken komité anmeldelsen skal sendes til se her.

Konkrete eksempler

Nedenfor gengives eksempler på praksis i komitésystemet om anmeldelsespligt.

Hvis man er i tvivl, om et projekt skal anmeldes til en videnskabsetisk komité, bør man indsende sagen til komiteen.

Forsøg med metode til differentiering af smagsstoffer i fødevareingredienser

Et forsøg havde til formål at finde en metode til at differentiere smagsstoffer i fødevareingredienser ved at registrere hjernens elektriske aktivitet (EEG) på bl.a. sucrose hos raske forsøgspersoner, hvor skiftevis tungens højre og venstre side blev stimuleret med ingredienserne.

Afgørende for, at komitéen fandt forsøget anmeldelsespligtigt var, at:

- forsøget inddragede levendefødte menneskelige individer, samt at
- man ville intervenere ved at undersøge de biologiske processer i hjernen i forbindelse med indtagelse af sødestoffer med formålet at skabe ny, generel viden om disse processer.

Der blev også lagt vægt på:

- Lovgivningen indeholder grænser for, hvilke fødevarer disse ingredienser må tilsættes og i hvilke mængder, for at indtagelsen heraf ikke medfører sundhedsrisiko

- I forbindelse med EEG-måling af hjernen kunne der opstå tilfældighedsfund med relevans for forsøgsdeltagerne

- Undersøgelser af, hvorvidt hjernen kan differentiere mellem fødevareingrediensers smagskarakteristika, som kan have sundhedsfremmende formål, fx fedmeforebyggelse eller diabetes.

Forsøg med mindfulness – anmeldelsespligt

Et forsøg havde til formål at undersøge, om de kognitive virkningsmekanismer i mindfulness-træning har effekt i forhold til arbejdshukommelse, opmærksomhed, beslutningstræning og stress. Der skulle indgå fysiske test og adfærdstest på raske forsøgspersoner (unge sportsudøvere).

Afgørende for anmeldelsespligt var:

- Forsøget inddragede levendefødte menneskelige individer
- Der var tale om en terapeutisk intervention med effektmål, der afspejlede, at man forventede klinisk effekt af interventionen på forskellige psykologiske og somatiske parametre.

Der blev også lagt vægt på:

- Mindfulness anvendes på nogle kliniske afdelinger som behandlingsmetode
- At der blev taget pulsmålinger og blodprøver.

Forsøg med genomsekventering af arkæologisk materiale i et demografisk studie

I et forsøg mente forskerne ud fra SNP chip-analyser at have konstateret, at den centralasiatiske befolkning i høj grad viste både europæisk, kaukasisk, sydsibirisk og

østasiatisk oprindelse. Man ville foretage komplet genomsekventering for at gennemføre en grundig demografisk undersøgelse. Herudover ønskede man at registrere områder af genomet, som er under naturlig selektion, og eventuelt bestemme regioner af genomerne, som har givet den centralasiatiske befolkning adaptive fordele til deres omgivelser. Der var indsamlet spytprøver fra nulevende personer af centralasiatisk oprindelse. Herudover ville man ”producere genom-dækkende data fra 140 forhistoriske personer”.

NVK vurderede, at projektet ikke var anmeldelsespligtigt med følgende begrundelse:

- Hovedformålet med projektet var at gennemføre demografiske undersøgelser samt undersøge hvilke genomer, der har givet den centralasiatiske befolkningsgruppe adaptive fordele til deres omgivelser

- Man ville anvende sundhedsvidenskabelig forskning til støtte for anden forskning, der ikke havde noget med medicinsk forskning at gøre

- Selv om der i forbindelse med studiet potentielt kunne opnås viden om den centralasiatiske befolknings biologiske udviklingsproces, var dette ikke hovedformålet med projektet

- Hovedformålet var dermed ikke at generere ny viden om sygdommes opståen eller om forebyggelse, diagnostik eller behandling af sygdomme

- Den blotte kortlægning af menneskets arvemasse (med mulighed for at skabe viden om biologiske processer) medførte ikke i sig selv, at der er tale om et anmeldelsespligtigt sundhedsvidenskabeligt forskningsprojekt

- Der kortlægges også arvemasse fra arkæologiske prøver, hvor beskyttelsesinteressen (i forhold til enkeltindividet og pårørende) ikke er den samme.
