# Supplementary material for: Novel sunprotection interventions to prevent skin cancer: A randomized study targeting Danes going on vacation to destinations with high UV index
Source: PLoS One. 2020 Dec 31;15(12):e0244597. doi: 10.1371/journal.pone.0244597 (PMC7774952; doi:10.1371/journal.pone.0244597)
Supplement: S3 File — (PDF) [file pone.0244597.s005.pdf]

DATATILSYNET

Privat forskning og statistik

Anmeldelse af behandlinger af oplysninger om personers rent private forhold, der foretages for en privat dataansvarlig, og som udelukkende finder sted i statistisk eller videnskabeligt øjemed. Der ansøges samtidig om Datatilsynets tilladelse.

Bemærk: Der skal *ikke* foretages anmeldelse til Datatilsynet, hvis ovennævnte behandlinger af personoplysninger sker i forbindelse med

- kliniske forsøg med lægemidler omfattet af lov om lægemidler,
- kliniske afprøvninger af medicinsk udstyr omfattet af lov om medicinsk udstyr,
- sundhedsvidenskabelige forskningsprojekter omfattet af lov om videnskabsetisk behandling af sundhedsvidenskabelige forskningsprojekter, eller
- behandling af personoplysninger, som foretages af studerende under arbejdet med projekt- og specialeopgaver mv. som led i deres erhvervsakademi-, professionsbachelor-, bachelor- eller kandidatuddannelse eller uddannelse på tilsvarende niveau, når behandlingen sker med udtrykkeligt samtykke fra den registrerede.

En anmeldelse til og tilladelse fra Datatilsynet vil således ikke kunne omfatte sådanne behandlinger.

1. Dataansvarlig og databehandlere	Dataansvarlig (f.eks. virksomhed, organisation, forening eller enkeltperson)<br>Brian Køster
	Evt. stilling (hvis enkeltperson er dataansvarlig)<br>Postdoc
	Adresse<br>Strandboulevarden 49, 2100, København Ø
	E-mail<br>brk@cancer.dk
	Tlf.nr.<br>35257666
	☐ Den dataansvarlige er etableret i et tredjeland, og der er udpeget en repræsentant i Danmark. Angiv navn og adresse på repræsentanten:

	☑ Der benyttes en eller flere databehandlere. Angiv navn(e) og adresse(r) på samtlige databehandlere:<br>Rambøll Management Consulting<br>Olof Palmes Alle 20<br>DK-8200 Aarhus N<br><br>**Bemærk:** Den dataansvarlige skal ikke også stå som databehandler. En databehandler er en ekstern part, som behandler oplysninger efter den dataansvarliges instruks og på den dataansvarliges vegne.
2. Betegnelse og formål	Projektet/undersøgelsens titel:<br>Test of developed interventions to prevent skin cancer: a randomized study targeting Danes going on vacation to sunny destinations with high UV index
	Formålet med projektet/undersøgelsen:<br>Formålet er at teste en række udviklede effektive interventioner målrettet Danskere der rejser på ferie til solrige destinationer. Det testes om interventionerne mindsker skoldning ved at øge brugen af skygge, hat, beskyttende tøjog solcreme for at forebygge kræft i huden i den danske befolkning.

3. Generel Beskrivelse	Følgende typer af behandling indgår:<br><br>Indsamling, registrering, opbevaring, analyse, anonymisering, sletning og eventuelt videregivelse.<br><br>Hvis der anvendes databehandlere, jf. ovenfor under pkt. 1, handler disse alene på den dataansvarliges vegne og efter instruks fra denne.<br><br>Der vil ikke blive behandlet flere oplysninger, end det er nødvendigt af hensyn til formålet med undersøgelsen.<br><br>Oplysningerne vil i videst muligt omfang blive behandlet i en form, hvor de ikke er umiddelbart personhenførbare, f.eks. i krypteret form eller under et løbenummer i stedet for under personnummer eller navn.<br><br>Behandling af personoplysninger vil ske under iagttagelse af persondataloven, herunder vilkår i Datatilsynets tilladelse.<br><br>Indsamlede oplysninger omfattet af persondatalovens § 10 vil alene blive behandlet med henblik på at udføre statistiske eller videnskabelige undersøgelser og vil ikke blive anvendt som grundlag for konkrete retlige eller faktiske foranstaltninger over for de registrerede eller andre personer. Oplysningerne vil således ikke blive anvendt i forbindelse med patientbehandling eller administrativ sagsbehandling.<br><br>Herunder vil der alene ske videregivelse til brug for andre undersøgelser, der udelukkende sker i statistisk eller videnskabeligt øjemed, og udelukkende efter forudgående indhentet tilladelse fra Datatilsynet, jf. persondatalovens § 10, stk. 2 og 3.
4. Kategorier af registrerede og oplysningstyper	Der behandles oplysninger om personer, der indgår i den videnskabelige eller statistiske undersøgelse.<br><br>Der vil kunne blive behandlet oplysninger om racemæssig eller etnisk baggrund, politisk, religiøs eller filosofisk overbevisning, fagforeningsmæssige tilhørsforhold, oplysninger om helbredsmæssige, seksuelle og strafbare forhold, oplysninger om væsentlige sociale problemer og andre rent private forhold, i det omfang sådanne oplysninger er relevante for undersøgelsen.
5. Modtagere	1) Eventuelle databehandlere, jf. pkt. 1.<br><br>2) Dataansvarlige for andre statistiske eller videnskabelige undersøgelser. Dette forudsætter særskilt, forudgående tilladelse fra Datatilsynet, jf. persondatalovens § 10, stk. 3.
6. Tredjelande	Angiv, om der vil ske overførsel af personoplysninger til lande uden for EU/EØS:<br><br>☒ Der påtænkes **ikke** overført personoplysninger til tredjelande.<br><br>☐ Der påtænkes overført personoplysninger til tredjelande på følgende grundlag:<br><br>☐ Der anvendes Kommissionens standardkontrakter uden ændringer (§ 27, stk. 4, jf. stk. 5).<br><br>☐ Oplysningerne overføres til et sikkert tredjeland, og der ansøges om Datatilsynets tilladelse til overførslen, jf. persondatalovens § 50, stk. 2 (gælder kun følsomme oplysninger omfattet af lovens §§ 7 og 8).<br><br>☐ Andet – beskriv:

	Overførsel til tredjelande sker med følgende formål:<br><br>Oplysningerne overføres til databehandler – angiv tredjelandet:<br><br>Andet – beskriv formålet med overførslen og angiv tredjelandet:
7. Sikkerhed	Der vil blive truffet sikkerhedsforanstaltninger i overensstemmelse med Datatilsynets standardvilkår for private forsknings- og statistikprojekter samt evt. supplerende vilkår fastsat af Datatilsynet. Standardvilkårene findes på www.datatilsynet.dk.
8. Påbegyndelse	Dato for påbegyndelse af behandlingen:<br>01.04.18
9. Sletning	Senest ved undersøgelsens afslutning, eller når statistikken er udarbejdet, vil personoplysninger – herunder eventuelt biologisk materiale – der behandles i forbindelse med den videnskabelige eller statistiske undersøgelse, blive slettet/destrueret eller anonymiseret, så det ikke længere er muligt at identificere de registrerede personer. Alternativt kan oplysningerne overføres til arkiv efter arkivlovens regler.

10. Udfyldt af	Dato*<br>12.02.18	Navn*<br>Brian Køster

Bemærk:

- Datatilsynets tilladelse skal foreligge, inden behandlingen iværksættes
- Ændringer skal meddeles Datatilsynet
- Den dataansvarlige skal selv opbevare en kopi af anmeldelsen
- De oplysninger, der indtastes i anmeldelsesblanketten, kan blive offentliggjort på Datatilsynets hjemmeside eller udleveret til interesserede.
